# Excitation-contraction coupling in mammalian skeletal muscle: Blending old and last-decade research

**DOI:** 10.3389/fphys.2022.989796

**Published:** 2022-09-02

**Authors:** Pura Bolaños, Juan C. Calderón

**Affiliations:** ^1^ Laboratory of Cellular Physiology, Centre of Biophysics and Biochemistry, Venezuelan Institute for Scientific Research (IVIC), Caracas, Venezuela; ^2^ Physiology and Biochemistry Research Group-PHYSIS, Faculty of Medicine, University of Antioquia, Medellín, Colombia

**Keywords:** skeletal muscle, excitation-contraction coupling, Ca^2+^, fluorescence, Ca^2+^ channels, ryanodine receptor—RYR1, mitochondria

## Abstract

The excitation–contraction coupling (ECC) in skeletal muscle refers to the Ca^2+^-mediated link between the membrane excitation and the mechanical contraction. The initiation and propagation of an action potential through the membranous system of the sarcolemma and the tubular network lead to the activation of the Ca^2+^-release units (CRU): tightly coupled dihydropyridine and ryanodine (RyR) receptors. The RyR gating allows a rapid, massive, and highly regulated release of Ca^2+^ from the sarcoplasmic reticulum (SR). The release from triadic places generates a sarcomeric gradient of Ca^2+^ concentrations ([Ca^2+^]) depending on the distance of a subcellular region from the CRU. Upon release, the diffusing Ca^2+^ has multiple fates: binds to troponin C thus activating the contractile machinery, binds to classical sarcoplasmic Ca^2+^ buffers such as parvalbumin, adenosine triphosphate and, experimentally, fluorescent dyes, enters the mitochondria and the SR, or is recycled through the Na^+^/Ca^2+^ exchanger and store-operated Ca^2+^ entry (SOCE) mechanisms. To commemorate the 7^th^ decade after being coined, we comprehensively and critically reviewed “old”, historical landmarks and well-established concepts, and blended them with recent advances to have a complete, quantitative-focused landscape of the ECC. We discuss the: 1) elucidation of the CRU structures at near-atomic resolution and its implications for functional coupling; 2) reliable quantification of peak sarcoplasmic [Ca^2+^] using fast, low affinity Ca^2+^ dyes and the relative contributions of the Ca^2+^-binding mechanisms to the whole concert of Ca^2+^ fluxes inside the fibre; 3) articulation of this novel quantitative information with the unveiled structural details of the molecular machinery involved in mitochondrial Ca^2+^ handing to understand how and how much Ca^2+^ enters the mitochondria; 4) presence of the SOCE machinery and its different modes of activation, which awaits understanding of its magnitude and relevance *in situ*; 5) pharmacology of the ECC, and 6) emerging topics such as the use and potential applications of super-resolution and induced pluripotent stem cells (iPSC) in ECC. Blending the old with the new works better!

## 1 Introduction

By the 1940s, some evidence was published considering the existence of a link between the excitation and the contraction of the skeletal muscle. However, it was very scarce, fraught with technical limitations and in some cases speculative, although considered “plausible”. Alexander Sandow stated in 1952 that the muscle “dependence of contraction on excitation indicates that there must be some process that is initiated in the excited membrane and which by moving inward makes contact with the contractile elements so as to initiate contraction. We shall designate the entire sequence of reactions -excitation, inward acting link, and activation of contraction- by the term excitation-contraction (E-C) coupling” (Sandow, 1952). This topic was so interesting by that time among researchers, that less than 20 years later the nature of the “inward acting link” was clear.

Since then, we have seen the development of a huge amount of technology with increasingly improved temporal and spatial resolution, which has been applied to gain insight into the excitation-contraction coupling (ECC) in skeletal muscle and have helped us outline the current landscape of this phenomenon.

Here, we present the basics of the ECC in skeletal muscle under physiological conditions, highlighting recent exciting conceptual advances and technical developments for its study. We present information gathered in vertebrate models but focused on mammalian ECC. Blending the old with the new allows us to commemorate 7 decades of hard work by honoring notable, classical contributions from many researchers in the field, but also by integrating new advances performed by intrepid young generations. We believe this is the best way to yield the most comprehensive possible picture of the ECC in skeletal muscle.

### 2 Ultrastructure, molecular machinery, and events of the excitation–contraction coupling mechanism in skeletal muscle

#### 2.1 Historical landmarks

The events responsible for the abrupt muscle transition from rest to contraction occupied famous physiologists back in the 40 s of the 20^th^ Century. Two well accepted concepts by that time were that the sarcolemma had excitatory properties (assumed to be limited only to the surface of the sarcolemma) and that soon after excited, the active state of the muscle was established (Kuffler, 1947; Hill, 1948; Hill, 1949). Moreover, there was a causal relationship because the kinetics of the contractile responses were modulated by the excitation (Kuffler, 1947; Sandow, 1952; Huxley and Taylor, 1958; Caputo, 2011). The activation seemed not to follow directly the excitation, because a longitudinal current and its associated electric field failed to induce a contractile response (Kuffler, 1947; Sten-Knudsen, 1954). An “inward acting link” (Sandow, 1952) between both, the excitation and the contraction, seemed thus necessary for many authors (Kuffler, 1947; Hill, 1948), mainly because they happen in parts of the cell separated by microns. Also, relevant information about a sizeable, yet manipulable, milliseconds delay between excitation and contraction, when several phenomena could be measured (Hill, 1949; Sandow, 1952; Weber and Portzehl, 1954), further suggested that a real link should exist. The duration of that window time was temperature-dependent, for instance, and there was a heat associated to activation which appeared after that latent period, before the shortening heat itself was measured (Hill, 1949; Sandow, 1952). A chemical or energetical link seemed feasible, but “to resolve its occurrence in times of the order of a few milliseconds would be one of fantastic difficulty” (Hill, 1949).

Simple ionic, radial diffusion from the sarcolemma caused by the longitudinal field of the action potential was not the link responsible for the activation of the whole fibre (Hill, 1948; Sten-Knudsen, 1954). Hill did his calculations with Ca^2+^ probably influenced by the results of Heilbrunn and Wiercinski (Heilbrunn and Wiercinski, 1947), who were “interested in knowing which substances will cause a shortening or contraction of the living protoplasm inside the muscle cell” (Heilbrunn and Wiercinski, 1947). According to their results, Ca^2+^ but neither Na^+^, K^+^, nor Mg^2+^, induced a notable muscle shortening when injected (Heilbrunn and Wiercinski, 1947).

It then became obvious that the link and its working mechanism was something else than the simple diffusion of a “contractile substance” released from the sarcolemma. Since the activation followed the points where the action potential passed (Sandow, 1952; Sten-Knudsen, 1954), the link should be a more complex process or may have a structural component. As most of the study models employed by that time were not mammalian, that structural element was associated to the Z lines at the I bands (Huxley and Taylor, 1958). Finishing the 50 s, and before the T-tubules (TT) and the triads were fully acknowledged (they were not unambiguously defined in the first images, and sometimes were thought to be part of the sarcoplasmic reticulum -SR-), the ECC was proposed to involve the excitation at the sarcolemma, then somehow the excitation penetration radially along a structure that, like the SR, should have a network nature mainly located at the Z lines. The resulting changes of charge density in those periodic places would lead to the release of Ca^2+^ from unknown resources (Csapo and Suzuki, 1957; Porter and Palade, 1957; Huxley and Taylor, 1958). This decade confirmed that Ca^2+^ was the activator of the contraction (Heilbrunn and Wiercinski, 1947; Weber and Portzehl, 1954; Niedergerke, 1955; Ebashi and Endo, 1968), probably by modulating the adenosine triphosphatase (ATPase) activity of the contractile machinery (Weber, 1959), but did not make clear what its origin was.

The latter model proved to be, in general terms, qualitatively correct when the 60 s well recognized the TT and its continuity with the sarcolemma (Endo, 1964; Franzini-Armstrong and Porter, 1964; Huxley, 1964) and the inward spread of the action potential through them (Ebashi and Endo, 1968). Also, the triad evolved from “two vesicles with the intervening space” (Porter and Palade, 1957) to the complex formed when the “T system is bordered on both sides by the terminal sacs of the SR” (Franzini-Armstrong and Porter, 1964). Subsequent work unveiled the main intracellular reservoir of Ca^2+^ and evidenced that the diffusion of Ca^2+^ starts when released from such reservoirs at periodic places which coincided with the terminal cisternae of the triadic regions (Hasselbach and Makinose, 1962; Huxley, 1964; Winegrad, 1965; Jöbsis and O’Connor, 1966; Ridgway and Ashley, 1967; Ashley and Ridgway, 1968; Ebashi and Endo, 1968; Winegrad, 1968; Winegrad, 1970), although the exact release mechanism was not fully understood. Huxley envisioned this in 1964 as: “It seems much more likely that the depolarization of the central element of the triad triggers off the release of calcium from the side elements … and that the specialized junctional regions seen are involved in this transmission process” (Huxley, 1964). The inward spread of excitation and the transient apparition of Ca^2+^ in the sarcoplasm constituted the long sought “inward acting link”. The discovery of a dual effect of adenosine triphosphate (ATP), which led to the proposal of a relaxing, “Marsh-Bendall” factor in muscle homogenates, and demonstrated to be the non-soluble, vesicle-located, ATP-Mg^2+^ activated sarcoendoplasmic reticulum Ca^2+^ pump (SERCA) (Kielley and Meyerhof, 1948; Marsh, 1951; Weber and Portzehl, 1954; Kumagai et al., 1955; Ebashi and Lipmann, 1962; Hasselbach and Makinose, 1962; Hasselbach, 1964; Ebashi and Endo, 1968; Caputo, 2011), closed the basic cycle of release and reuptake of Ca^2+^ from and to the SR.

Finally, Ebashi and Endo (Ebashi and Endo, 1968), who identified the molecular link between the sarcoplasmic Ca^2+^ and the activation of the contractile machinery, and also participated in the “relaxing factor” work, delineated the basic ECC sequence pretty much as we know it now: “The processes which would bring the contractile elements to the active state may be listed as follows: Action potential and its inward spread through the T-system … the sarcoplasmic reticulum is the site of linkage between excitation and contraction, i.e., Ca ion associated with a certain part of the sarcoplasmic reticulum is released by the influence of the electrical current field, induced by depolarization of the surface membrane, and Ca ion thus released subsequently activates the contractile system”, then, the “sarcoplasmic reticulum, exerts its relaxing effect by removing Ca ion from the contractile system *in vivo*”. Saul Winegrad complemented: “it is likely that … the longitudinal tubules and the intermediate cisternae … contain the calcium-sequestering system that is believed to operate during relaxation. The calcium taken up by these structures presumably then moves more slowly to the terminal cisternae, the main storage site in the muscle which has completely recovered from mechanical activity” (Winegrad, 1968).

Successive research enriched that sequence with structural and functional details, mainly pertaining to the specific issue of the Ca^2+^ release from the triad and added regulators and more precise quantitative data to the whole process. Also, demonstrated that all these events are extremely coupled. The readers are referred to the Historical Compendium of Muscle Physiology, for further historical details (Caputo, 2011).

#### 2.2 The sequence of events and the molecular machinery involved in the excitation–contraction coupling

The ECC mechanism in skeletal muscle depicts a fast communication between electrical events taking place in the sarcolemma and the muscle contraction, through a cascade of global and locally restricted Ca^2+^ transients. The sequence of events entails: 1) initiation and propagation of an action potential (AP) along the plasma membrane, 2) inward, radial spread of the depolarization along the tubular system, 3) dihydropyridine receptors (DHPR)-mediated sensing of changes in the membrane potential, 4) allosteric interaction of the DHPR with the SR Ca^2+^ release channels (ryanodine receptors, RyR), 5) rapid release of Ca^2+^ from the triadic regions of the SR and transient increase of Ca^2+^ concentration ([Ca^2+^]) in the myoplasm, 6) transient activation of the contractile apparatus and the myoplasmic Ca^2+^ buffering system, 7) activation of the membranous-linked Ca^2+^ buffering and transporting system, which comprises the SERCA and the Na^+^/Ca^2+^ exchanger (NCX), and 8) appearance of the cascade of secondary mitochondria-restricted and tubular-restricted Ca^2+^ transients, the latter reflecting the recycling of Ca^2+^ through the store-operated Ca^2+^ entry (SOCE) mechanism.

Physiologically, the activation of the muscle fibre is modulated by the depolarization of the sarcolemma, including the tubular system (Kuffler, 1947; Hodgkin and Horowicz, 1960a). Under resting conditions, the fibre is polarized between –70 (Head, 1993) and –83 mV (Luff and Atwood, 1972; Wang et al., 2022), at 22 and 37°C, respectively. Upon binding of acetylcholine (ACh) to the motor end plate, the inward sarcolemmal conductance to Na^+^ rapidly increases, bringing about an AP. In most experiments, performed between 15 and 30°C, the AP depolarizes the fibre and then slightly polarizes it to positive values between +25 and +35 mV, which seems to be a safety factor for a successful AP conduction and SR Ca^2+^ release (Wang et al., 2022). The AP spike has 1.5–2.5 ms of duration at half-maximum amplitude and spreads along the sarcolemma over both sides of the motor plate with a propagation velocity of 0.4–1.9 m/s, depending on the measurement technique, the temperature of the experiment and the muscle studied (Luff and Atwood, 1972; Delbono and Stefani, 1993; DiFranco et al., 2008; Pedersen et al., 2011; Banks et al., 2018). The tension-sarcolemmal potential relationship is sigmoidal, with a threshold for activation at about −54–58 mV (Hodgkin and Horowicz, 1960a; Caputo, 2011).

During its sarcolemmal travel, the AP enters sarcolemmal invaginations known as TT. These are periodic, radially (on a transversal section) or transversally (on a longitudinal section) oriented membranous structures (80–100 × 35–40 nm wide), which conduct the AP at about 1 cm/s in a Na^+^ dependent, regenerative way (Huxley, 1964; González-Serratos, 1971; Bezanilla et al., 1972; Edwards et al., 2012). The so-called TT are actually a tubular network which comprises transverse (∼75%), diagonal (∼10%) and longitudinal (∼15%) tubules (Huxley, 1964; Jayasinghe et al., 2013; Jayasinghe and Launikonis, 2013). This structural arrangement, as well as its electrical properties, secures the rapid delivery of the AP to the interior of the fibre (Bezanilla et al., 1972; Fraser et al., 2011; Pedersen et al., 2011; Edwards et al., 2012), a crucial step for the uniform release of Ca^2+^ and the subsequent rapid contraction.

The incoming excitation reaches the triadic regions, where a TT is surrounded by two radially dilated portions of the SR, called “terminal cisternae” (Porter and Palade, 1957; Franzini-Armstrong and Porter, 1964; Huxley, 1964). The junctional parts of the terminal cisternae (jSR), and the TT, of the triadic regions, house a bunch of proteins involved in the regulation of the release of Ca^2+^ from the SR. The central actors in this process are the DHPR anchored to the TT and the RyR1 anchored to the SR membrane, which constitute the Ca^2+^ release units (CRU). DHPR (L-type Ca^2+^ channel, Ca_V_1.1) are heteropentamers formed by subunits α_1_ (transmembrane), γ (transmembrane), β_1_ (intracellular), α_2_ (extracellular), and δ_1_ (extracellular), whose function is regulated by the membrane potential. The cryoelectron microscopy (cryo-EM) reconstructions at 2.7–3.6 Å confirmed that the α_1_ subunit of the channel has the typical 6 × 4 structure of many voltage-gated channels, i.e., four homologous domains (DI-IV), each with six transmembrane helices (Wu et al., 2016; Zhao et al., 2019). The α_1_ subunit houses the dome, the pore domain (PD), the selectivity filter (SF), and the voltage-sensing domain (VSD). The dome is a negatively charged, progressively narrowing region mainly shaped by extracellular loops above the SF of the PD, to which it guides Ca^2+^. The PD is made up of the S5, S6 and P helices of each domain, and several loops stabilized by multiple disulfide bonds, which create a permeation path for Ca^2+^ of about 60 Å in length. The SF is a specialized, narrow region, predominately formed by negatively charged aminoacids: E292 and G293 of DI, E614 and D615 of DII, E1014 and G1015 of DIII, and E1323 and A1324 of DIV. Since N617 of DII seems also to be particularly important for Ca^2+^ permeation (Dayal et al., 2017; Idoux et al., 2020; Dayal et al., 2021), the SF is likely more complex than initially proposed (Wu et al., 2016). The auxiliary subunits have a regulatory role on the expression, localization and function of the channel (Gregg et al., 1996).

Since the DHPR carries a Ca^2+^ current under voltage-clamp protocols in intact fibres (Skoglund et al., 2014; Dayal et al., 2017; Banks et al., 2021), it is expected to function the same as a response to an AP, highlighting its nature as a voltage-gated channel. The activated Ca^2+^ inward current is slower, and with a slightly lower amplitude compared to the Ca_v_1.2 present in the heart, however, the influx of Ca^2+^ through this channel is not necessary for the skeletal muscle ECC and contraction (Caputo and Gimenez, 1967; Armstrong et al., 1972; Dayal et al., 2017; Idoux et al., 2020). Instead, the ability of the DHPR to sense the AP is particularly important for the skeletal muscle ECC. The S4 transmembrane helices of the α_1_ subunit constitute the voltage sensors (VSDI to VSDIV), which decode the information of the tubular excitation and translates it into a signal for the RyR1. The voltage sensing function depends on the S4 enrichment in the positively charged aminoacids arginine and lysine. Their movement during the VSD operation produces a small, yet measurable, voltage-dependent intramembrane charge movement, i.e., a current, which precedes the activation of the Ca^2+^ release from the SR (Schneider and Chandler, 1973; Rios and Brum, 1987; Banks et al., 2021). The peak of the charge movement time course follows the peak of the AP by 1.5 ms (Banks et al., 2021). It is intriguing why there are sizeable differences in the amplitudes, voltage-dependence and time courses of the VSDI-IV movements (Banks et al., 2021; Savalli et al., 2021), and whether they actually tune in any way the Ca^2+^ release from the SR. For instance, the VSDII and VSDIV seem to be the first ones activated, but the VSDI is so slowly activated that it seems not to be directly involved in the Ca^2+^ release activation; contradictory results have been reported regarding the activation kinetics of VSDIII (Banks et al., 2021; Savalli et al., 2021). In any case, the activation of either one or several of the DHPR´s VSD likely leads to a conformational change that gates the opening of the RyR1 in a cooperative way (Schneider and Chandler, 1973; Rios and Brum, 1987; Ríos et al., 1993).

Functional experiments with molecularly engineered DHPR suggested that its loop DII-III, close to the VSDII, is fundamental to ECC (Tanabe et al., 1990). However, the 3D structures available just lack the region between residues 687–789 (Wu et al., 2016; Zhao et al., 2019), which corresponds to the loop DII-III, precluding a conclusion about if it is long enough to clearly reach and interact with the RyR. If not directly, this loop may still interact with the RyR through the SH3 and cysteine-rich domain containing (STAC3) protein (Rufenach and Van Petegem, 2021; Shishmarev et al., 2022), something which awaits to be confirmed as a step to prove if STAC3 mediates the DHPR-RyR coupling relevant for a successful ECC.

Alternatively, the loop I-II-AID-β_1_ complex (Wu et al., 2016), also close to the VSDII, protrudes from the DHPR to the myoplasm and may directly or indirectly (i.e., the discovery of accessory β_1_-binding proteins such as Rem opens this possibility) interact with the RyR, explaining early functional observations according to which the absence of the β_1_ subunit eliminates the ECC (Gregg et al., 1996; Beqollari et al., 2015).

The history has shown that solving this issue is particularly difficult, but to fully understand the ECC mechanism, it is necessary first to make clear if, and how, the DHPR gates the RyR1 through a direct DHPR-RyR interaction or requires one or several accessory proteins (e.g., STAC3, Rem). Afterwards, it is crucial to have the complete, atomic resolution structure of the DHPR-RyR or DHPR-accessory proteins-RyR complexes under different conformations, to unambiguously assign the domains that mediate their interaction and shed light on the gating mechanism. Using purified complexes or native membranes would be, at least theoretically, possible approaches to address this problem.

Supramolecularly, the DHPR are arranged in groups of four, called tetrads, which alternately face the highly ordered RyR1, filling the 15–25 nm TT-SR gap (Huxley, 1964; Block et al., 1988; Franzini-Armstrong and Jorgensen, 1994; Franzini-Armstrong et al., 1998; Franzini-Armstrong, 1999).

The RyR1 are mushroom-like (from its lateral view), four-leaf clover shaped (from the sarcoplasmic view), Ca^2+^ channels with a big cytoplasmic moiety and a transmembrane region, inserted in rows in the jSR (Fleischer et al., 1985; Imagawa et al., 1987; Block et al., 1988; Saito et al., 1988). The effort of many laboratories worldwide, and the gain in resolution in cryo-EM recent reconstructions (between 3.8 and 6.1 Å) (Efremov et al., 2015; Yan et al., 2015; Zalk et al., 2015), compared to the first structures (over 9 Å) (Block et al., 1988; Saito et al., 1988; Wagenknecht et al., 1989; Ludtke et al., 2005; Samsó et al., 2005), reached a point at which a model of six transmembrane segments for each of the four monomers (6 × 4) that ensemble the functional channel appears reasonable. Thus, the transmembrane region of each monomer looks alike other ion channels: four transmembrane α-helices (S1 to S4) surround the S5 and S6 pore-forming helices. The luminal loops, the S6 and the P-segments constitute an extended permeation pathway of about 80 Å in length, which includes a 10 Å long SF, which drains into a 15 Å long hydrophobic cavity. A motif enriched in glycine residues of the S6 along this pathway is particularly important for gating and Ca^2+^ permeation in this channel (Efremov et al., 2015; Mei et al., 2015; Yan et al., 2015; Zalk et al., 2015).

The cytoplasmic moiety comprises about 80% of the bulk of the protein and is a complex network of tens of α-helices, surrounding one central spot (SPRY domains) enriched in β-sheets, which conform up to 20 domains (Efremov et al., 2015; Chen and Kudryashev, 2020), mainly involved in binding and transducing the signaling of many ligands to the pore region. Among those domains rich in α-helices, the EF-hand motifs, and the repeat 3-4 highlight: the EF-hands are in the lower face of the cytoplasmic moiety, while the repeats 3-4 are on top and at the corners of the cytoplasmic moiety, where they are involved in Ca^2+^ sensing and DHPR-RyR interaction, respectively. These structures seem to be the responsible for two putative modes of activation of the RyR1: mediated by the DHPR in those RyR1 coupled to tetrads, and by Ca^2+^ in those RyR1 not coupled to tetrads. From the nice images published (Samsó et al., 2009; Efremov et al., 2015; des Georges et al., 2016), we can say that the conformational change observed during opening of the channel resemble the flowering of a rose: a central twist and dilation, accompanied by a notorious change in the periphery of the structure which move outwards and downwards. This seems to be associated with an increase in curvature in the SR membrane when observed in native membranes by cryo-electron tomography (Chen and Kudryashev, 2020).

Upon opening, the RyR1 allows a rapid, massive, highly regulated release of Ca^2+^ from the SR to the myoplasm. From the peak of the AP, the peak of the release of Ca^2+^ is delayed by about 2–3 ms in most experiments performed between 15 and 25°C (Delbono and Stefani, 1993; Banks et al., 2021). This time window encompasses the charge movement in the DHPR, the RyR1 gating and opening, and the Ca^2+^ diffusion from the terminal cisternae to the myoplasm. Although differences among fibre types have been recognized (Section 3.1), the peak of the release of Ca^2+^ is attained within 1.8 ms in most fibres (Calderón et al., 2009; Calderón et al., 2010; Calderón et al., 2014a; Calderón et al., 2014b; Rincón et al., 2021). Beyond the DHPR, a handful of endogenous regulators of the Ca^2+^-release function of the RyR1, acting either from the myoplasmic or the SR luminal side, have been described: ATP and other purines, Ca^2+^, Mg^2+^, reactive oxygen species (ROS) and reactive nitrogen species, redox state, phosphorylation/dephosphorylation status, calmodulin, S100A1, FK 506 binding protein 12 (FKBP12 or calstabin-1), triadin (Trisk-95 and Trisk-51), junctin, homer-1, calumenin-2 and calsequestrin (CASQ) (Imagawa et al., 1987; Lai et al., 1988; Hidalgo et al., 2005; Butanda-Ochoa et al., 2006; Jung et al., 2006; Wei et al., 2006; Goonasekera et al., 2007; Feng et al., 2008; Prosser et al., 2008; Wei et al., 2009; Boncompagni et al., 2012; Wium et al., 2012; Marty, 2015; Meissner, 2017; Ogawa et al., 2021; Woll and Van Petegem, 2022).

Although the SR protein-27 (SRP-27) and junctophilin (JPH)-1 interact with the RyR1 (Phimister et al., 2007; Bleunven et al., 2008), it is not clear yet if they actually regulate the channel. There is debate on whether JPH-2 interacts or not with RyR1 (Phimister et al., 2007; Nakada et al., 2018). The ability of JPH-1 and -2 to regulate Ca^2+^ release in myotubes likely relies on their DHPR-binding ability and their TT-jSR tethering properties, which also mediate the precise localization of CRU (Nakada et al., 2018; Perni, 2022).

First isolated from rabbit muscle, CASQ is a ∼44-kDa Ca^2+^ binding protein highly expressed in the lumen of the jSR (MacLennan and Wong, 1971; Franzini-Armstrong et al., 1987; Perni et al., 2013), where it undergoes a [Ca^2+^]-dependent cooperative and reversible polymerization, forming oligomers with both low and high affinity sites for Ca^2+^ (Park et al., 2003; Sanchez et al., 2012). When [Ca^2+^] approaches 1 mM, the three thioredoxin-similar domains which surround a hydrophilic core fold, exposing numerous negatively charged aspartate and glutamate residues, that stack front-to-front forming dimers, which in turn stack back-to-back and continue stacking as [Ca^2+^] increases, to form a ribbon-like polymers that can ramify as a tree, finally forming a mesh with multiple nodes (Wang et al., 1998; Park et al., 2003; Park et al., 2004; Sanchez et al., 2012; Kumar et al., 2013; Perni et al., 2013; Wang and Michalak, 2020). Those “branches” anchor directly, or through triadin and junctin, to the RyR1, forming a complex that modulates the SR Ca^2+^ release (Guo and Campbell, 1995; Zhang et al., 1997; Wei et al., 2006; Goonasekera et al., 2007; Wei et al., 2009; Boncompagni et al., 2012; Sanchez et al., 2012; Wang and Michalak, 2020). Its ability to bind up to 80 ions per molecule explains why CASQ1 keeps the total SR Ca^2+^ as high as 35–175 mM (Royer and Ríos, 2009; Wang and Michalak, 2020). The electrostatic binding of Ca^2+^ to CASQ, together with its low affinity sites, favors the rapid unbinding and release of Ca^2+^.

The apparition of Ca^2+^ in the myoplasm shows microdomains with an up to 20-fold gradient of [Ca^2+^], which depends on the distance of a subcellular region from the CRU (Escobar et al., 1994; Baylor and Hollingworth, 2007; DiFranco et al., 2008; Hollingworth et al., 2012; Holash and MacIntosh, 2019). The average of those variable Ca^2+^ microdomains generates a global, positive, myoplasmic Ca^2+^ transient. The amount of Ca^2+^ released from the SR is enough to rise the resting cytoplasmic free [Ca^2+^] from 45–106 nM (Williams et al., 1990; Westerblad and Allen, 1991; Head, 1993; Konishi, 1998) to a fibre-type dependent averaged value of 7–30 µM (Hollingworth et al., 1996; Baylor and Hollingworth, 2003; Baylor and Hollingworth, 2007; Hollingworth et al., 2012; Milán et al., 2021; Rincón et al., 2021) (Section 3.1). In turn, this global, master Ca^2+^ transient associates to a cascade of locally restricted Ca^2+^ transients, from which a diversity of phenomena is activated (contraction, metabolism, heat, etc), such as the positive mitochondrial Ca^2+^ transients, and the negative SR and tubular Ca^2+^ transients. Also, as soon as the Ca^2+^ appears in the myoplasm, several Ca^2+^ buffering mechanisms are activated. Troponin C (TnC), parvalbumin (PV), ATP and the Ca^2+^ indicators rapidly bind Ca^2+^. Each of these Ca^2+^ binding mechanisms produces its own Ca^2+^ transient. Subsequently, the NCX, the mitochondria and the SERCA deal with Ca^2+^ with slower kinetics and the SOCE machinery recycles part of the Ca^2+^ extruded through the NCX. Each mechanism will be further developed in the coming paragraphs.

Troponins are a family of proteins attached to the thin filaments, from which TnC binds several Ca^2+^ ions with moderate affinity and are the molecular link between the cytosolic Ca^2+^ raise and the activation of the contraction (Ebashi et al., 1969). The beginning of the contraction shows a delay of 2–3 ms with respect to the beginning of the Ca^2+^ release, at room temperature, reflecting the diffusion time and the binding to TnC.

Muscle PV is a low molecular weight protein of the ɑ-sublineage (ɑ-PV), particularly abundant in muscle fibres type II, in which it can reach ∼1,000 µM. Two, high affinity, EF-hand, Ca^2+^ binding sites, are the responsible for its role as a Ca^2+^ buffer important in muscle relaxation, as it was recognized long time ago (Gillis et al., 1982; Heizmann et al., 1982; Leberer and Pette, 1986; Füchtbauer et al., 1991; Permyakov and Uversky, 2022). The binding of Ca^2+^ to TnC and PV explains most of the heat produced during muscle activation but is PV the responsible of the heat absorption observed several milliseconds after the Ca^2+^ release (Barclay and Launikonis, 2021). Given its differential concentration, and its high affinity when present, PV also has an important role in shaping the different morphologies of the single and tetanic Ca^2+^ transients obtained in different fibre types (Calderón et al., 2014a) (Section 3.1).

ATP reaches concentrations even higher than PV, which give it importance as a Ca^2+^ buffer, however, its lower Ca^2+^ affinity gives it less total capacity for Ca^2+^ binding than TnC and PV (Baylor and Hollingworth, 2003; Rincón et al., 2021). Under experimental conditions, the Ca^2+^ dyes also buffer Ca^2+^, within a variable range of kinetics that depend on the concentration and the intrinsic properties of the indicator molecule (Section 3.1).

Once the unbound Ca^2+^ returns to the myoplasm, relaxation proceeds. Ca^2+^ is definitively removed by the mechanisms responsible to extrude it from the myoplasm. The first mechanism activated is the NCX. This protein is located mainly in the tubular network, where it extrudes Ca^2+^ from the sarcoplasm, with a low total capacity and rapid saturation. Given this kinetics, its role is more evident during tetanic than during single stimulation (Balnave and Allen, 1998; Calderón et al., 2014a; Rincón et al., 2021). Part of this extruded Ca^2+^ is recycled back to the myoplasm during and after each twitch through Orai1 and likely the transient receptor potential canonical (TRPC) channels, reflecting a phasic activation of the SOCE mechanism (Section 3.3). This NCX-SOCE coupling likely reflects part of the bidirectional SR-TT exchange of Ca^2+^ proposed back in the 70 s of the last Century (Winegrad, 1968; Winegrad, 1970).

The uptake of Ca^2+^ by the mitochondria also removes Ca^2+^ from the myoplasm. The mitochondrial Ca^2+^ transients follow the cytosolic one with a ∼10 ms delay. Actively studied during the last two decades, at least two lines of evidence suggest that its sizeable buffering capacity shapes the cytosolic Ca^2+^ transient helping the muscle relax, further carrying metabolic consequences. On one side, dual mitochondrial and myoplasmic Ca^2+^ measurements demonstrated larger cytosolic Ca^2+^ transients in regions with polarized vs. depolarized mitochondria in intact flexor digitorum brevis (FDB) fibres during a single twitch, which allowed to estimate a rather large capacity of 10–18% to buffer the cytosolic Ca^2+^ transient (Yi et al., 2011). On the other side, poisoning different fibre types with FCCP (Section 3.4) induced a reversible lengthening of the decay phase of single and tetanic Ca^2+^ transients (Caputo and Bolaños, 2008; Calderón et al., 2014a). Recent knowledge about the fine structure of the molecular machinery involved in Ca^2+^ transport into the mitochondria, as well as the determination of the precise amount of Ca^2+^ released from the SR and the peak Ca^2+^ concentration reached in the sarcoplasm, allows us now to describe how and how much Ca^2+^ enters the mitochondria during ECC in different fibre types (Section 3.2).

The final mechanism responsible for restoring the sarcoplasmic resting [Ca^2+^] and keeping it low is the SERCA. SERCA is a high molecular weight, highly regulated pump, enriched in the longitudinal region of the SR and the non-junctional membrane of the terminal cisternae, which transports Ca^2+^ into the SR against its concentration gradient (Hasselbach, 1964; Jorgensen and Jones, 1986; Hasselbach, 1998; Odermatt et al., 1998; Periasamy and Kalyanasundaram, 2007; Rathod et al., 2021). This protein has three large cytoplasmic domains, N, P and A, attached to a domain consisting of 10 hydrophobic trans-SR-membrane helices (M1 to M10) (MacLennan et al., 1985; Toyoshima and Mizutani, 2004). ATP and Mg^2+^ binding to the more peripheral N domain activates its large movement towards the more central P domain and the turning of the A domain over itself. The resulting movements of some transmembrane helices, particularly M1, M2 and M4, occlude two Ca^2+^ ions within the transmembrane region and then release them inside the SR (Toyoshima and Mizutani, 2004). Even when a single Ca^2+^ transient is only ∼4–12 ms width, depending on the type of the fibre, SERCA pumping remains active for more than 60 ms, while dealing with the Ca^2+^ 1) being unbound from the TnC and PV, 2) leaving the mitochondria and 3) entering the fibre *via* SOCE.

**FIGURE 1:**
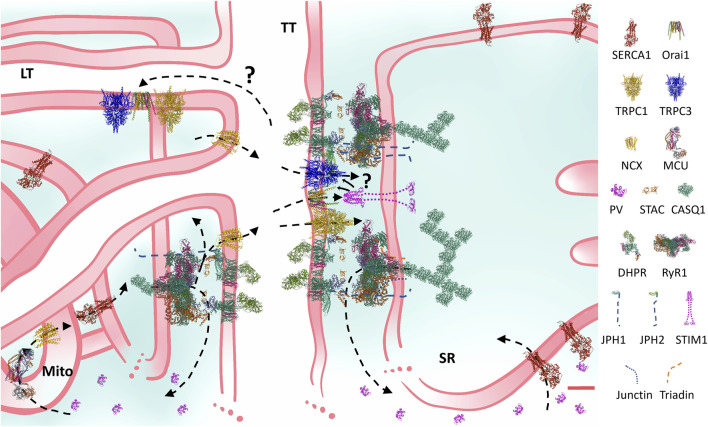
Structural and functional comprehensive model of the Ca^2+^ handling machinery and Ca^2+^ fluxes of the mammalian skeletal muscle ECC. Constructed at scale based on microscopy and protein measurements, the cartoon considers many current observations and addressed the question if there is space for all ECC proteins in the triadic space. For instance, JPH-1 coprecipitates with RyR1 more than JPH-2, also, super-resolution observations suggest that JPH-1 locates more closely to RyR1 than JPH-2. Regarding the SOCE machinery, some TRPC members have been shown to precipitate with DHPR and Orai. The model shows that a TRPC-Orai-TRPC cluster explaining these observations fits among DHPR-RyR1-empty squares. STIM location and size enables its binding to the TRPC-Orai-TRPC cluster. Also, this pattern may explain how TRPC may be a mediator of the RyR1 involvement in SOCE regulation. It is clear that preassembled STIM-Orai clusters present a solution to the problem of severe movement restrictions in this region. Arrows indicate Ca^2+^ fluxes. The internal equilibrium of Ca^2+^ entails short (on the right: sarcoplasmic reticulum (SR) release, myoplasmic buffering and SERCA reuptake) and long (on the left: SR release, myoplasmic and mitochondrial (Mito) buffering and activation, and SERCA uptake) routes. The external equilibrium entails the Ca^2+^ exit through the NCX and the entry through the SOCE channels, located in the transverse (TT), and probably in the longitudinal tubules (LT). Question marks indicate probable routes or mechanisms, which have not been “seen” yet. Although the high amount of PV was meant to represent a type II fibre, in general terms, the structure and the direction of the fluxes presented remain qualitatively the same in all fibre types. In the few cases in which the structure of the muscle isoforms have not been determined, homologous were used (e.g., non-mammalian NCX or STIM1 were used instead of NCX1-3 or STIM1L). Key to the figure: ECC: excitation-contraction coupling; SOCE: store-operated Ca^2+^ entry; SERCA: sarcoendoplasmic reticulum Ca^2+^ adenosine triphosphatase; TRPC: transient receptor potential canonical; NCX: Na^+^/Ca^2+^ exchanger; MCU: mitochondrial Ca^2+^ uniporter; PV: parvalbumin; STAC: SH3 and cysteine-rich domain containing protein; CASQ: calsequestrin; DHPR: dihydropyridine receptor; RyR: ryanodine receptor; JPH: junctophilin; STIM: stromal interaction molecule. Protein data bank structures: 1RTP, 1VFP, 2K60, 2MAJ, 3J8H, 3TEQ, 5GJV, 5JDG, 5KN1, 5ZBG, 6BBF, 6K7Y, 6UY7, 7RW4, 7RXQ. Red calibration bar: 10 nm.

The last decade was particularly fruitful to take to the next level observations performed in the “old”, past century: 1) researchers finally obtained structures of the DHPR and RyR at near-atomic resolution, helping explain the gating of both channels and mapping several regulators; 2) the structural and functional importance of the new main triadic regulators (JPH, triadins, junctin and STAC3) was acknowledged; 3) the peak myoplasmic [Ca^2+^] was reliably quantified (Section 3.1); 4) evidence consolidated the importance of mitochondria in Ca^2+^ handling in skeletal muscle (Section 3.2); 5) the ECC is ultimately a cascade of global and restricted Ca^2+^ transients associated to the fibre excitation whose concerted action activates the contractile machinery and other functions of the skeletal muscle. Figure 1 presents a complete, updated structural and functional model of the ECC in mammalian skeletal muscle.

## 3 Focus on

Here, we will focus on selected topics which have received particular attention during the last decade and whose results can be articulated with previous observations to reach stronger conclusions.

### 3.1 Assigning reliable numbers to the Ca^2+^ fluxes and concentrations in mammalian muscle fibres

How much Ca^2+^ is released from the SR and with which kinetics? Which free [Ca^2+^] is reached in the myoplasm? Is this Ca^2+^ enough to activate contraction? What is the kinetics of the Ca^2+^ reuptake? These questions have been addressed for at least 5 decades, when pioneer work demonstrated the transient increase in myoplasmic Ca^2+^ associated to the membrane depolarization in amphibia and arthropods muscles using murexide and aequorin as Ca^2+^ indicators (Jöbsis and O’Connor, 1966; Ridgway and Ashley, 1967; Ashley and Ridgway, 1968; Ebashi and Endo, 1968). The researchers then focused on amphibia until the early 90 s (Gillis et al., 1982; Baylor and Hollingworth, 1988; Caputo and Bolaños, 1994; Escobar et al., 1994), and finally moved to mammalian muscles.

Three attainments coincided at the time at which the field focused on Ca^2+^ measurements in mammalian muscle models: 1) availability of relevant biochemical information on the ECC and fibre types, 2) the outburst of fluorescent Ca^2+^ indicators and 3) improvements in calibration, mathematical and computational modelling.

Here, we will focus on how researchers integrated these three attainments to finally find a solution to the challenge of putting reliable numbers to the ECC Ca^2+^ fluxes and concentrations in mammalian muscle, especially considering the existence of at least four fibre types.

### 3.1.1 Biochemical information relevant to the mammalian excitation–contraction coupling in fibre types

The existence of muscles with different biochemical and dynamic properties was formally acknowledged long time ago (Ranvier, 1873; Close, 1972). The study of their particularities first focused on the biochemical differences of their fibres (Dubowitz and Pearse, 1960; Engel, 1962; Brooke and Kaiser, 1970; Barnard et al., 1971; Peter et al., 1972). By the 90s, a wealth of information confirmed the presence of at least four phenotypes in the muscles of the mammalian extremities, as recognized by the presence of the isoforms of the myosin heavy chain (MHC): I (slow twitch), and IIA, IIX/D and IIB (fast twitch) (Schiaffino and Reggiani, 2011).

Soon after the ECC phenomenon was demonstrated to be mediated by Ca^2+^, many researchers presented data concerning biochemical differences in the molecular machinery involved in Ca^2+^ release and reuptake using the dichotomic model of slow-twitch vs. fast-twitch fibres. Later, the studies were extended to the four fibre types. Details about the molecular and biochemical differences that underlie the quantitative differences in ECC among fibre types have been presented previously and we refer the readers to those papers and the literature cited therein (Bottinelli and Reggiani, 2000; Calderón et al., 2010; Calderón et al., 2014b; Rincón et al., 2021).

We can summarize the most relevant information as follows: 1) there are between twofold and threefold more CRU in the fast, compared to the slow fibres, with no difference in the isoforms. There are only about 1.5 times more triadic accessory proteins (e.g., triadin, JPH) in the fast compared to the slow muscles. Fast fibres only express CASQ1, while slow fibres have both CASQ1 and CASQ2 at a 3:1 ratio, but with a total amount of CASQ, and a total Ca^2+^ buffering capacity, somewhat lower than in fast fibres. Despite this, there seems to be only a ∼10% difference in SR free Ca^2+^ content between both types of fibres; 2) TnC isoforms differ between slow and fast fibres, explaining the presence of almost twofold more Ca^2+^ binding sites in fibres types IIA, IIX/D and IIB, compared to type I; 3) there is a continuum increase in PV content across the four fibre types such that the fastest fibres (type IIB) have up to 300 times more than the slowest fibres (type I). Fibres IIA have about tenfold more PV than type I; 4) the differential ATP content explains the about 50% higher amount of ATP Ca^2+^ binding sites in the fast fibres compared to slow ones; 5) SERCA different isoforms and content among fibres result in a twofold (for IIA) or up to fivefold (for IIX/D and IIB) larger maximum Ca^2+^ reuptake flux rate in fast fibres compared to fibres type I; 6) up to twofold higher mitochondrial volume and a larger maximum flux rate of the mitochondrial Ca^2+^ uniporter (MCU) explain the threefold to fourfold higher capacity of this mechanism in fibres type I compared to fibres type II; 7) NCX1 is more abundant in fibres type I, but the capacity of the NCX3, present in fibres type II, is slightly higher; 8) STIM1 is about 1.5 times more abundant in slow compared to fast fibres, however, the SOCE total capacity seems to be higher in the latter.

Short (FDB) and large (extensor digitorum longus, EDL, and soleus) fibres, either in fascicles, manually isolated or enzymatically dissociated, intact or nude, have been the most used models to study Ca^2+^ kinetics in different fibre types. Dynamical and molecular markers have been used to identify different fibre types in those preparations (Baylor and Hollingworth, 2003; Calderón et al., 2009; Calderón et al., 2010; Hollingworth et al., 2012; Calderón, 2013; Calderón et al., 2014a).

### 3.1.2 Using Ca^2+^ dyes that reliable track the Ca^2+^ transients

Foremost researchers popularized the use of fluorescent intracellular dyes for determining the dynamic concentration of intracellular Ca^2+^ in different cell types (Tsien, 1980; Tsien et al., 1982; Grynkiewicz et al., 1985; Minta et al., 1989). A variety of dyes was then developed and many used in skeletal muscle (Raju et al., 1989; Delbono and Stefani, 1993; Hollingworth et al., 1996; Gee et al., 2000; Katerinopoulos and Foukaraki, 2002; Woods et al., 2004) (Table 1). It became clear soon that slow dyes well measured resting Ca^2+^ but did not render trustable measurements of peak [Ca^2+^] and Ca^2+^ kinetics (Baylor and Hollingworth, 1988; Berlin and Konishi, 1993; Hollingworth et al., 1996; Wokosin et al., 2004). This is because of their slow rate of detachment from Ca^2+^ and their low Ca^2+^ *Kd* (below 1 µM *in vitro*), limiting the range of concentrations at which the dye responds before significantly buffering Ca^2+^ and becoming saturated. Moreover, the calibration of non-ratiometric, slow dyes is fraught with difficulties, for instance, large errors in the F_min_ estimations are common, and they may induce up to a 17% error in the [Ca^2+^] (Mejía-Raigosa et al., 2021). As a result, a wealth of qualitatively relevant information was generated by using these slow dyes, however, quantitative reliable information was still lacking.

**TABLE 1:** Best Ca^2+^ dyes to study ECC in skeletal muscle, with their affinity and rate constants relevant for calibration of their fluorescence signals.

Dye	*K*_*d*_ (µM)	*k*_*on*_ (µM^−1^ s^−1^)	*k*_*off*_ (s^−1^)	*K*_*d*_ (µM)	*k*_*on*_ (µM^−1^ s^−1^)	*k*_*off*_ (s^−1^)	Cellular model	T (°C)^b^	Comments	References
	*In vitro*			*In situ* ^a^						
High affinity Ca^2+^ dyes (*K*_*d*_ *in vitro* < 1 µM)^c^										
Calcium Green-1	0.19			0.93			HeLa cells	20–22	*In vitro* and *in situ K*_*d*_ were measured	Thomas et al. (2000)
Calcium Orange	0.19			1.10			HeLa cells	20–22	*In vitro* and *in situ K*_*d*_ were measured	Thomas et al. (2000)
Fluo-3	0.33–0.51	920	424	0.81–4.00	13.1–15	33.5–60	Frog intact muscle fibres, HeLa cells	16–22	*In vitro* values were measured. *In situ* values were either measured using 55 mg/ml of aldolase to simulate intracellular conditions or estimated.	Minta et al. (1989), Hollingworth et al. (1990), Lattanzio and Bartschat, (1991), Harkins et al. (1993), Caputo and Bolaños, (1994), Gee et al. (2000), Thomas et al. (2000)
Oregon Green 488 BAPTA-1	0.16–0.17			0.43			HeLa cells	20–22	*In vitro* and *in situ K*_*d*_ were measured	Thomas et al. (2000), Woods et al. (2004)
Fluo-4	0.345			1.00			HeLa cells	20–22	*In vitro* and *in situ K*_*d*_ were measured	Gee et al. (2000), Thomas et al. (2000)
Fura-2	0.14–0.24	270–760	65–109	0.23	100	23	Frog intact muscle fibres	16–24	*In vitro K*_*d*_ was measured, *k*_*on*_ and *k*_*off*_ values were either measured or estimated. *In situ* values were estimated	Grynkiewicz et al. (1985), Baylor and Hollingworth, (1988), Lattanzio and Bartschat, (1991), Berlin and Konishi, (1993)
Intermediate affinity Ca^2+^ dyes (1 µM < *K*_*d*_ *in vitro* < 2 µM)										
Fura-4F	1.16							20–21	*In vitro K*_*d*_ was measured	Wokosin et al. (2004)
Rhod-2	1.00							22	*In vitro K*_*d*_ was measured	Minta et al. (1989)
Low affinity Ca^2+^ dyes (*K*_*d*_ *in vitro* > 2 µM)										
Calcium Green 5N	63–85			156	6.4	1,000	Frog intact muscle fibres	16–22	*In vitro K*_*d*_ was measured. *In situ* values were estimated	Zhao et al. (1996)
Calcium Orange 5N	53–55			87	12	1,040	Frog intact muscle fibres	16–22	*In vitro K*_*d*_ was measured. *In situ* values were estimated	Zhao et al. (1996)
Fluo-5N	90			350			Rat skinned muscle fast fibres	21–24	*In vitro* and *in situ K*_*d*_ were measured	Cully et al. (2016), Gee et al. (2000)
Mag-Fura-2	44–58.5	125–233	5,875–11,416	100	>50	>5,000	Mammalian and frog skeletal muscle fibres	16–24	*In vitro K*_*d*_ was measured, *k*_*on*_ and *k*_*off*_ were estimated. *In situ* values were estimated from frog data.	Berlin and Konishi, (1993), Delbono and Stefani, (1993), Zhao et al. (1996), Wokosin et al. (2004), Baylor and Hollingworth, (2011)
Mag-Fluo-4^d^	7.25 × 10^3^ µM^2^			1.65 × 10^5^ µM^2^	5.28 μM^−2^ s^−1^	8.72 × 10^5^	Type II FDB intact muscle fibres from mouse	20	*In vitro* and *in situ K*_*d*_ were measured. *In situ k*_*on*_ and *k*_*off*_ were estimated. *In vitro k*_*on*_ and *k*_*off*_ were not estimated since they are irrelevant for calibration of *in situ* Ca^2+^ fluorescence signals.	Milán et al. (2021)
Mag-Fura-5	23–31					>5,000	Rat cut fast muscle fibres and frog muscle fibres	16–22	*In vitro K*_*d*_ was measured. *In situ k*_*off*_ was estimated	Delbono and Stefani, (1993), Zhao et al. (1996), Szentesi et al. (1997)
Mag-Fura-red	55			242	>21	>5,000	Frog intact muscle fibres	16–22	*In vitro K*_*d*_ was measured. *In situ* values were estimated	Zhao et al. (1996)
Magnesium Green	7		1,750	19	90	1,750	Frog intact muscle fibres		*In vitro K*_*d*_ was measured. *In situ* values were estimated	Zhao et al. (1996)
Oregon Green 488 Bapta-5N	48	1.57	7,520					22	*In vitro* values were measured	Woods et al. (2004)
Rhod-5N				800	6.25	>5,000	Rat skinned fast fibres	21–24	*In situ K*_*d*_ was measured, *k*_*on*_ and *k*_*off*_ were estimated	Cully et al. (2016)

a Although *in vitro* and *in situ* values are given for comparative purposes, only *in situ* values should be used for calibration of sarcoplasmic Ca^2+^ signals. The “Cellular model” column applies only to *in situ* values.

b Temperature applies to both *in vitro* and *in situ* values.

c All *K*_*d*_ values are for the Ca^2+^ **─**dye reaction. High and intermediate affinity dyes should only be used for qualitative studies (e.g., the phenomenon is present or not), relative and comparative studies (e.g., a percentage change compared to a control condition) or resting Ca^2+^ assessment. Low affinity dyes can be used for absolute quantifications of Ca^2+^ transients, comparative measurements, and gathering data for feeding mathematical models.

d Since Mag-Fluo-4 has a 2:1 (dye:Ca^2+^) stoichiometry, the units of the *K*_*d*_ and *k*_*on*_ are different from the other dyes, as indicated in the table. Regrettably, due to a typing error, the *K*_*d*_ *in vitro* was originally reported as 7.25 × 10^5^, being 7.25 × 10^3^ the correct number. FDB: flexor digitorum brevis.

Fast Ca^2+^ dyes such as Fluo-5N, Mag-Fluo-4, Mag-Fura-2 (Furaptra) and Rhod-5N, typically have an *in vitro Kd* between 20 and 100 µM, which rise to about 700–1,000 µM *in situ*, i. e, in the myoplasm or in a medium resembling the myoplasm. This very low affinity *in situ* is the property that makes them reliably track Ca^2+^ kinetics in skeletal muscle, as demonstrated because these dyes resolve every single peak of a high-frequency tetanus, and their fluorescence signals superimpose the actual Ca^2+^ transients (Baylor and Hollingworth, 2003; Baylor and Hollingworth, 2007; Calderón et al., 2014a; Rincón et al., 2021). Moreover, this property makes them be far from saturation, be less prone to buffer the Ca^2+^ transients, and during calibration, their F_min_ is less susceptible to errors because their fluorescence in presence of the resting [Ca^2+^] is already very low (Hollingworth et al., 1996; Baylor and Hollingworth, 2003; Milán et al., 2021).

Of the above mentioned, polycarboxylate, fast dyes Mag-Fura-2 and Mag-Fluo-4 are the most trustable to gain quantitative insight into the ECC in different fibre types (Table 1). Mag-Fura-2 was originally developed as a ratiometric Mg^2+^ dye, with a similar spectra as that of Fura-2, but it also binds Ca^2+^ with low affinity, and a 1:1 (Ca^2+^:dye) stoichiometry (Raju et al., 1989). For Ca^2+^ measurements, it has been typically excited between 350 and 430 nm, and its emission has been collected over 480 nm, either as ratiometric or as non-ratiometric (Hollingworth et al., 1996; Baylor and Hollingworth, 2003). An *in situ Kd* of 98 µM was estimated at 16°C (Hollingworth et al., 1996; Zhao et al., 1996), but experimental measurements in mammalian fibres are pending. Mag-Fluo-4 is a Fluo-4 derivate, with a 1:2 stoichiometry, with excitation and emission peaks at 493–494 and 515–516 nm, respectively, and a good dynamic range (Gee et al., 2000; Milán et al., 2021). The Mag-Fluo-4 *in situ Kd* is 1.65 × 10^5^ μM^2^, measured in fast mice fibres at 20°C, which ensures that, even under short loading times (10 min), less than 5% of the dye is bound to Ca^2+^ during a twitch, thus being far from saturation (Milán et al., 2021). This dye binds more heavily than Mag-Fura-2 (74 vs. 54% of the indicator molecules bound) to intracellular components.

Given their structure, an apparent drawback of both dyes is the possibility of contamination of the Ca^2+^ signals with Mg^2+^. With a *Kd*_*Mg*_ *in vitro* of 5,300 µM for Mag-Fura-2, and a range of *Kd*_*Ca*_ of 44–58.5 µM (Table 1) at ∼20°C, a *Kd*_*Mg*_ */Kd*_*Ca*_ ratio of ∼90–120 is obtained (Hollingworth et al., 2009). Assuming a *Kd*_*Mg*_ of 6,500 µM (Baylor and Hollingworth, 2011) and a half-fluorescence concentration for Ca^2+^ of 31.5 µM (Milán et al., 2021), the *Kd*_*Mg*_ */Kd*_*Ca*_ for Mag-Fluo-4 at ∼20°C is ∼200. Since similar ratios may apply to *in situ* conditions, and given a resting free [Mg^2+^] below 1 mM (Westerblad and Allen, 1992), a significant contamination of the Ca^2+^ signals with Mg^2+^ in muscle fibres can be ruled out.

### 3.1.3 Employing a trustable calibration method

The truthful conversion of fluorescence signals into Ca^2+^ comprises the use of equations and values for parameters of affinity and fluorescence acquired *in situ* at similar temperatures (Table 1). Several equations have been published for ratiometric and non-ratiometric dyes, as well as for 1:1 or 1:2 stoichiometry (Grynkiewicz et al., 1985; Hollingworth et al., 1996; Zhao et al., 1996; Mejía-Raigosa et al., 2021), including the calibration of Mag-Fura-2 (Hollingworth et al., 1996; Baylor and Hollingworth, 2003) and Mag-Fluo-4 (Milán et al., 2021). For calibrating fast dyes, saponin is a better membrane permeabilizer than ionomycin (Milán et al., 2021).

### 3.1.4 Then, how much Ca^2+^ do muscle fibres move?

Two different groups have been devoted to put numbers to the mammalian ECC regarding fibre types, satisfactorily taking into account the three aspects discussed above, and have obtained similar results (Hollingworth et al., 1996; Baylor and Hollingworth, 2003; Baylor and Hollingworth, 2007; Calderón et al., 2009; Calderón et al., 2010; Baylor and Hollingworth, 2011; Calderón et al., 2011; Hollingworth et al., 2012; Calderón et al., 2014a; Calderón et al., 2014b; Milán et al., 2021; Rincón et al., 2021). Their data, acquired using Mag-Fura-2 and Mag-Fluo-4, can be pooled, and presented as the following statements: 1) fibres type I and IIA share the Ca^2+^ transient kinetics called morphology type I (MT-I), while the fibres IIX/D and IIB share the morphology type II (MT-II); 2) single twitch MT-I signals have rise times between 1.2 and 1.8 ms and decay times of up to 80 ms. They release Ca^2+^ at rates between 50 and 150 μM/ms, and their peak sarcoplasmic free [Ca^2+^] ranges from 7 to 13 µM; 3) single twitch MT-II signals typically have rise times between 1.0 and 1.3 ms, with decay times ranging from 13 to 25 ms, being ∼3–4 times narrower than the MT-I signals; 4) MT-II fibres release Ca^2+^ at a huge rate of 200–250 μM/ms, and their peak sarcoplasmic free [Ca^2+^] ranges from 15 to 30 µM. For the sake of comparison, the peak SR release rate in mammalian cardiomyocytes ranges from 2.0 to 4.2 μM/ms (Song et al., 1998; Shannon et al., 2000); 5) the total amount of Ca^2+^ released from the SR in the MT-II is about 350 µM, ∼2.7 times higher than the amount released by the MT-I and about 5 times the amount released in cardiomyocytes (Song et al., 1998). The differential kinetics of the Ca^2+^ release partially explain the differential kinetics of the contraction in all fibre types (Calderón et al., 2010).

The variability in the values presented above reflects the inherent variability of the skeletal muscle biochemistry and function (Bottinelli and Reggiani, 2000; Bottinelli, 2001), its plasticity, as well as temperature (usually between 16 and 23°C) and sarcomere length differences between papers. Importantly, MT-I values almost never overlap with those of MT-II. Furthermore, these numbers reflect that the skeletal muscle fibre is the cell that deals with the largest and fastest release and reuptake of Ca^2+^, which, instead of its shortening ability, can be considered its main specialization.

The above numbers have fed increasingly complex mathematical models which have allowed to assign numbers to different compartments and Ca^2+^ binding mechanisms. A recent comprehensive model simulated the changes in Ca^2+^ concentrations and fluxes through the sarcomere of the four fibre types, considering classical (Tn, PV, ATP, SERCA, and dye) and new (mitochondria, NCX, and SOCE) Ca^2+^ binding sites, during single and tetanic stimulation, using Mag-Fluo-4 data (Rincón et al., 2021). The magnitudes of change of the Ca^2+^-bound forms of the Ca^2+^ buffers studied follow the order IIB ≥ IIX > IIA > I, except for the mitochondrial peak [Ca^2+^], which showed the pattern I >> IIA >> IIX ≥ IIB. The kinetics for fibres IIA and IIX proved to be intermediate between I and IIB fibres, supporting dynamic data (Bottinelli et al., 1991; Bottinelli and Reggiani, 2000; Bottinelli, 2001; Rincón et al., 2021).

An important issue is that the peak [Ca^2+^] described above agree well with several previous observations. For instance, the pCa_50_ of the Ca^2+^-induced superprecipitation reactions usually ranged from 5.9 to 5.1 at 23°C, i.e., ∼1–10 µM, in presence of physiological [Mg^2+^]. Similar observations were done regarding the tension-pCa relationship in skinned fibres, in which the maximum tension required a pCa ∼5.5─5.0, i.e., 3.2─10 µM in all fibre types (Ebashi and Endo, 1968; Bottinelli and Reggiani, 2000). Furthermore, the full activation of the contractile machinery and the appearance of mitochondrial Ca^2+^ transients *in vivo* requires [Ca^2+^] about one order of magnitude higher than 1–2 µM, which is the [Ca^2+^] that gives the 50% activation of these mechanisms (Ebashi et al., 1969; Sembrowich et al., 1985). The Ca^2+^ release from loaded SR vesicles, the open probability of the RyR1 and the ryanodine (Ry) binding kinetics, consistently show that the RyR1 half-activates at ∼1–5 µM and peaks between 10 and 30 µM Ca^2+^ at room temperature (Nagasaki and Kasai, 1983; Fill et al., 1990; Meissner, 2017). Finally, Ca^2+^ releases and fluxes as large as those reported above are required to account for the heat released during muscle activation, as recently demonstrated (Barclay and Launikonis, 2021).

In conclusion, although a differential biochemical data suggested different Ca^2+^ transient kinetics for the fibre types, and although biophysical evidence suggested an expected value for the peak sarcoplasmic [Ca^2+^] over 5 µM, it took a long way to finally assign reliable numbers to this issue: fibres type I and II have peak sarcoplasmic [Ca^2+^] between 7 and 13 µM, while fibres type IIX/D and IIB have values between 15 and 30 µM. The release rate and the total amount of Ca^2+^ released in fibres type IIX/D and IIB is ∼2–3 times larger than in fibres type I and IIA. Articulation of old data with data gathered during the last decade has made that coherence becomes now evident across biochemical (e.g., protein isoforms, reaction rates, dependence on Ca^2+^), dynamical (e.g., tension-pCa relationships) and biophysical (e.g., fluorescence, Ca^2+^ concentrations and fluxes) measurements and estimations.

### 3.2 Mitochondria in excitation–contraction coupling

Mitochondria are double membrane organelles, important regulators of cellular Ca^2+^ homeostasis, signaling, metabolism and energy production in the form of ATP, for which they have been named “the powerhouse of the cell” (Siekevitz, 1957) and “the hub of cellular Ca^2+^ signaling” (Szabadkai and Duchen, 2008). Moreover, they are highly dynamic, forming networks inside the cells and remodeling their morphology and activity (Anderson et al., 2019).

Recently, several reviews on the skeletal muscle mitochondria dynamics, with strong structural approaches have been published (Anderson et al., 2019; Bloemberg and Quadrilatero, 2019; Li et al., 2020; De Mario et al., 2021; Gherardi et al., 2021; Garbincius and Elrod, 2022). We also reviewed previously their importance for skeletal muscle (Calderón et al., 2014b). However, recent estimates of the rate of increase of Ca^2+^ and mitochondrial Ca^2+^ transients in different fibre types provide novel interesting quantitative information, not acknowledged in previous reviews, that fosters us to contribute this section. Thus, here we will center on the research relevant to understand the Ca^2+^ movements and concentrations into the skeletal muscle mitochondria and their relationship with ECC.

#### 3.2.1 Location and dynamics

**FIGURE 2:**
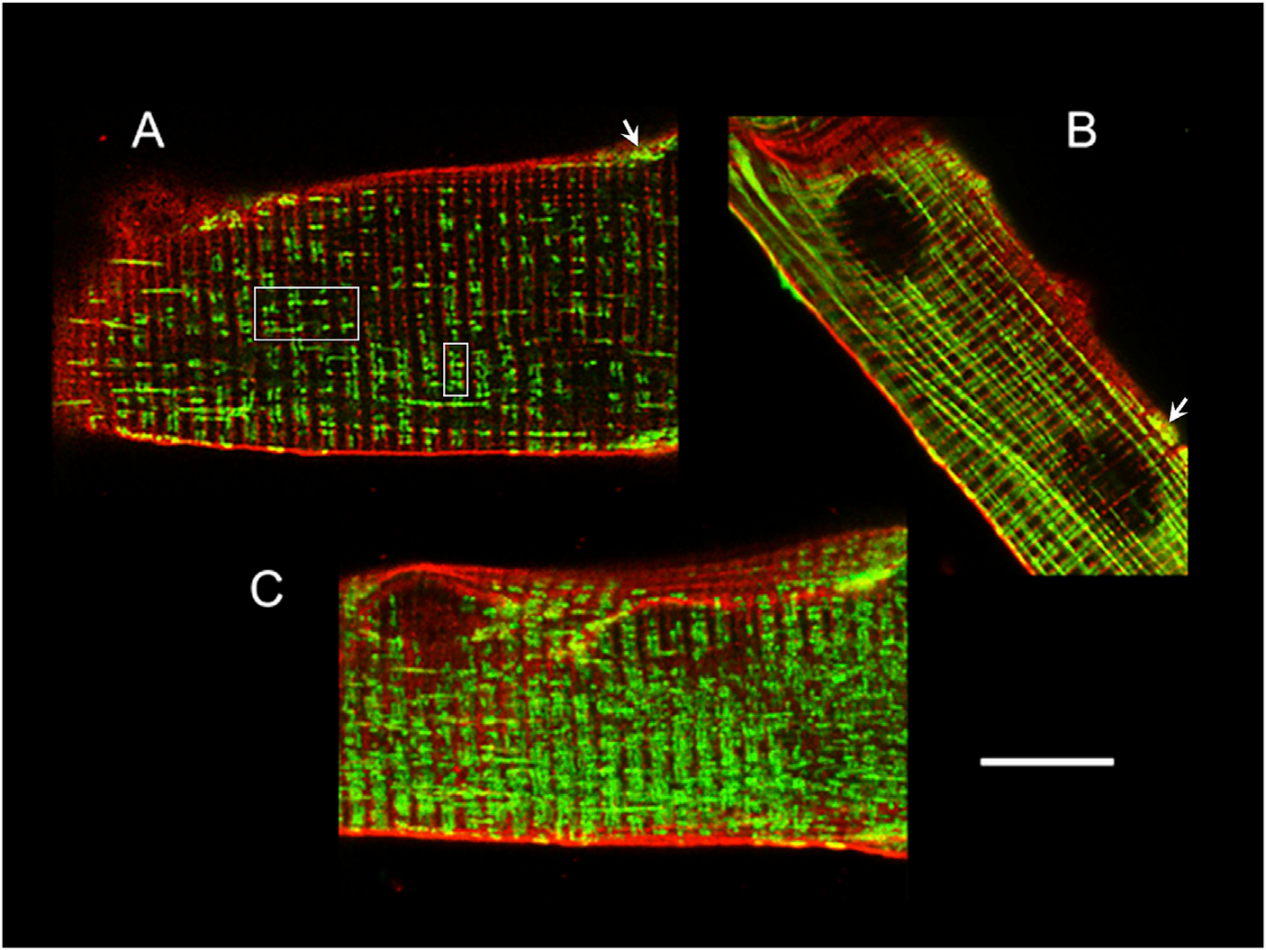
Mitochondria location and distribution in muscle fibres. Confocal images of adult mouse FDB fibres loaded with Di-8-Anneps (red) and Mitotracker Green (green) to stain membranes and mitochondria, respectively. The fibre end in **(A)** shows the T-tubules and intermyofibrillar mitochondria, either paired at both sides of the Z line near the center of the fibre (white squares) or forming elongated structures, which are much more evident in **(B)**, and look like columns parallel to the long axis of the fibre. The arrows in **(A)** and **(B)** point to typical clustered, less ordered, subsarcolemmal mitochondria. The mitochondrial network appearance is clearer under the 3D reconstruction shown in **(C)**. Calibration bar: 10 µm, applies to all panels.

The mitochondria inside the mammalian skeletal muscle fibre can be classified as: subsarcolemmal, intermyofibrillar and perinuclear. While the subsarcolemmal and perinuclear have certain mobility, the movements of the intermyofibrillar are more restricted. The first are clustered and less ordered, whilst the latter are packed between the contractile proteins and the SR membranes, or highly ordered within the I-bands by pairs at either side of the Z line, close to the terminal cisternae of the triads, forming a quasi-crystalline structure (Ogata and Yamasaki, 1997; Vendelin et al., 2005; Kuznetsov et al., 2006; Bolaños et al., 2009; Franzini-Armstrong and Boncompagni, 2011; Boncompagni et al., 2020) (Figure 2).

Tethers anchor the outer mitochondrial membrane (OMM) to the terminal cisternae opposite to the jSR (Bolaños et al., 2008; Dirksen, 2009b; Boncompagni et al., 2009; Pietrangelo et al., 2015). Those tethers, previously found in liver cells (Mannella et al., 1998; Csordás et al., 2006), are 10 nm long electron-dense structures (Dirksen, 2009b; Boncompagni et al., 2009; Pietrangelo et al., 2015), whose nature remains under debate. They may correspond to the ERMES (Endoplasmic Reticulum-Mitochondria Encounter Structure) complex found in budding yeast (Kornmann et al., 2009), formed by four components (Mmm1, Mdm10, Mdm12, and Mdm34) and several accessory proteins (e.g., Emr1), whose malfunction affects mitochondrial morphology (Rasul et al., 2021). They have also been associated with Mitofusin2 (Mfn2) (de Brito and Scorrano, 2008), whose absence reduces mitochondrial Ca^2+^ uptake (Ainbinder et al., 2015). This topic awaits further research.

Although previously considered isolated organelles, mitochondria can communicate among them in different cell types (Huang et al., 2013; Lavorato et al., 2017; Vincent et al., 2017; Lavorato et al., 2020). In skeletal muscle by means of fusion-fission, remodeling events or “kissing junctions”, they form elongated structures with narrow connecting ducts, and less frequently nanotunnels, acting as an independent and highly dynamic network which connects the matrixes of non-adjacent mitochondria (Vincent et al., 2016; Vincent et al., 2017; Vincent et al., 2019; Lavorato et al., 2020; Rahman and Quadrilatero, 2021). There has also been described synapses-like structures between adjacent mitochondria (Picard, 2015) that would help integrate information about the network (Picard et al., 2015). Those synapses, connecting ducts and nanotunnels will favor the communication between mitochondria anchored to the SR and the whole network in skeletal muscle and may also provide the structural basis to support the idea of enhanced propagation of intracellular signals (Díaz-Vegas et al., 2019). The “Excitation-Metabolism Coupling” (EMC) term has been put forward to integrate the coupling between the depolarization and the metabolic signaling through the mitochondrial network interconnexions (Díaz-Vegas et al., 2019). If these connecting structures and network somehow directly feedback the ECC, besides having a metabolic role, is an avenue that should be studied in the future.

In a different context, the EMC was previously used referring to the bidirectional communication between SR and mitochondria. Orthograde when the Ca^2+^ influx activates ATP synthesis and metabolism. Retrograde given that the ATP is also used for the SERCA to reuptake Ca^2+^ into SR as well as for the inhibition of local SR Ca^2+^ release regulation. EMC would be more important in mitochondria-enriched slow- and fast-twitch oxidative muscle than in fast-twitch glycolytic muscle (Dirksen, 2009b; Rossi et al., 2009).

Although the structural evidence of the mitochondrial network seems convincing, functional studies demonstrating their importance for the ECC are still lacking.

#### 3.2.2 How and how much Ca^2+^ enters the mitochondria?

The intermyofibrillar mitochondrial distribution confers them with a privileged position within ∼150 nm from the CRU of the jSR (Boncompagni et al., 2009) (Figure 2). Even in the case of a single twitch or caffeine exposure, mitochondrial Ca^2+^ can follow, with a short delay, the time course of the cytoplasmic Ca^2+^ increase (Rudolf et al., 2004; Shkryl and Shirokova, 2006; Bolaños et al., 2009; Yi et al., 2011; Karam et al., 2017), demonstrating that the myoplasmic Ca^2+^ increase observed during ECC is sensed by the mitochondria.

Given that the mitochondrial affinity for Ca^2+^ is intermediate, 1.2 µM for slow-twitch and 2 µM for fast twitch fibres (Sembrowich et al., 1985), and the first reported sarcoplasmic peak [Ca^2+^] were misleadingly low (even below 2 µM), it seemed difficult to explain how Ca^2+^ entered the mitochondria. However, as discussed (Section 3.1), during the last decade it became clear that all fibres reach between 7 and 30 µM free myoplasmic [Ca^2+^] just after excitation, making obvious that these values are at least one order of magnitude over the affinity of the mitochondria. Moreover, in compartmentalized models, larger free [Ca^2+^] between 40 and 60 µM can be attained just between the triads and the Z lines (Baylor and Hollingworth, 2007). This sizeable increase in Ca^2+^ close to the mitochondria, the higher sensitivity given by the skeletal muscle MICU1.1 variant (Section 3.2) and the large negative mitochondrial potential (ΔΨm, −180 mV) generate a strong electrochemical gradient which favors the diffusion and the Ca^2+^ transport into the mitochondrial matrix. The maximum flux rate was lately estimated to be 18.2 μM/s for fast and 74.3 μM/s for slow fibres (Rincón et al., 2021). A recent model showed for the first time that the mitochondrial Ca^2+^ transients reach 0.3 µM in fibres IIX/D and IIB, 0.5 µM in fibres IIA and 1.2 µM in fibres type I (Rincón et al., 2021).

The Ca^2+^ increase in the mitochondrial matrix enhances the ATP production necessary for muscle contraction, by activating the ATP synthase and enzymes of the tricarboxylic acid cycle and the oxidative phosphorylation (Kavanagh et al., 2000; Finkel, 2011). It also helps shaping the decay phase of the sarcoplasmic Ca^2+^ transient (Calderón et al., 2014a), likely speeding up the muscle relaxation. Depending on its kinetics, the excess of accumulated Ca^2+^ can also activate excessive ROS production and programmed dead of the cell through the opening of the mitochondrial permeability transition pore (mPTP) (Biasutto et al., 2016; Li et al., 2020).

#### 3.2.3 Recent advances in Ca^2+^ handling machinery in mitochondria

The Ca^2+^ influx from the cytoplasm to the mitochondrial intermembrane space (IMS) occurs through the porine-like, voltage-dependent anion channels (VDAC) placed in the OMM (Colombini, 1980; Colombini, 1983; Colombini, 2012; Shoshan-Barmatz and De, 2017; Shoshan-Barmatz et al., 2018). Out of the three isoforms present in mammalian cells (VDAC1, 2, 3) (Shoshan-Barmatz et al., 2010; Messina et al., 2012; Shoshan-Barmatz and De, 2017), VDAC1 is the most expressed in skeletal muscle (Massa et al., 2000). At low transmembrane voltage the channel is open with high conductance for anions allowing the diffusion of anionic metabolites and adenine nucleotides, being selective for ATP. However, at higher transmembrane voltage the channel close for anions and becomes selective for Ca^2+^.

From de IMS to the mitochondrial matrix the Ca^2+^ goes through channels inserted in the inner mitochondrial membrane (IMM): the MCU complex (Kirichok et al., 2004), the Rapid mode (RaM) (Sparagna et al., 1995) and the mRyR1 (Beutner et al., 2001; Ryu et al., 2011).

The MCU is a highly Ca^2+^ selective channel holocomplex at IMM (Baughman et al., 2011; De Stefani et al., 2011; De Stefani et al., 2016; Mammucari et al., 2016). This complex contains the MCU, the EF-hand Ca^2+^-binding proteins Mitochondrial Calcium Uptake 1 (MICU1) and MICU2 forming dimers and the Essential MCU Regulator (EMRE) (Sancak et al., 2013). MICU1 acts as a Ca^2+^-sensing gatekeeper, keeping the channel closed when Ca^2+^ levels are low and allowing the channel to open in response to transient rises (Perocchi et al., 2010; Mallilankaraman et al., 2012; Csordás et al., 2013; Plovanich et al., 2013; Sancak et al., 2013). In addition, MICU1 is also involved in maintaining the cristae structure and IMM anchorage of MCU (Gottschalk et al., 2019). The MCUb subunit acts as a dominant-negative subunit that reduces the MCU activity (Raffaello et al., 2013).

In skeletal muscle, MICU1.1, a spliced variant of MICU1, forms the heterodimers MICU1.1-MICU2 giving a higher affinity for Ca^2+^ (Vecellio Reane et al., 2016; Gherardi et al., 2021). Normally, MCU current densities in skeletal muscle reach up to 58 pA/pF (Fieni et al., 2012).

The third channel-forming subunit recently described is EMRE, a transmembrane protein of 10 kDa with a single IMM transmembrane domain (Sancak et al., 2013). EMRE is required for the interaction of MCU with MICU1.1 in skeletal muscle and MICU2. It is essential for *in vivo* uniporter current given that MCU oligomers alone are not sufficient for *in vivo* uniporter activity (Sancak et al., 2013). EMRE-dependent regulation requires MICU1.1, MICU2, and cytoplasmic Ca^2+^; its acidic C-terminal domain functions as a matrix Ca^2+^ sensor that regulates the MCU activity (Vais et al., 2016). Thus, EMRE acts together with MICU1 as a gatekeeper complex regulating the Ca^2+^ movements through MCU, able to sense Ca^2+^ at both sides of IMM and preventing mitochondria both from Ca^2+^ depletion and overload (Vais et al., 2016).

The Ca^2+^ release from the mitochondria is under the control of the mitochondrial Na^+^/Ca^2+^ exchanger (mNCX, NCLX) (Palty et al., 2010; Palty and Sekler, 2012; Garbincius and Elrod, 2022), the Ca^2+^/H^+^ antiporter (mNCH or LETM1 or mHCX) (Jiang et al., 2009; Tsai et al., 2014) and the mPTP (Li et al., 2020; Bernardi et al., 2021). The first two mechanisms, present in the IMM, are capable of reverse function (Garbincius and Elrod, 2022). The Ca^2+^ efflux by NCLX is slower than the Ca^2+^ influx by MCU (Rudolf et al., 2004) which may favor Ca^2+^ overload if cytoplasmic Ca^2+^ is much increased or NCLX diminished. The nature of the third mechanism, the mPTP, is still unknown, though recently it was proposed to be formed by a Ca^2+^-dependent conformation of the F-ATP synthase (Li et al., 2020; Bernardi et al., 2021). Physiologically, through transient short openings (flickerings), mPTP may regulate Ca^2+^ in the mitochondrial matrix (Hüser and Blatter, 1999; Petronilli et al., 1999; Li et al., 2020; Bernardi et al., 2021). Mitochondrial Ca^2+^ overload, likely reflecting an imbalance between the Ca^2+^ uptake and release mechanisms, triggers uncontrolled mPTP opening causing the loss of ΔΨm, impairing ATP production, increasing ROS and eventually leading to cell apoptosis (Biasutto et al., 2016; Li et al., 2020; Bernardi et al., 2021), an effect also seen with some protonophores which release mitochondrial Ca^2+^ (Bolaños et al., 2008; Caputo and Bolaños, 2008). A physiological ECC, associated to regular mitochondrial Ca^2+^ transients, is essential to keep mPTP closed, preventing these deleterious events (Li et al., 2020). Unfortunately, despite this interesting finding, the topic of the Ca^2+^ release from muscle mitochondria remains largely unaddressed. The kinetics of this phenomenon, its relationship with the whole ECC machinery, and eventual quantitative or qualitative differences among fibre types should be addressed in the future.

It is satisfactory to see that the last decade finally unveiled the structure of the molecular machinery and the mechanisms involved in the substantial transport of Ca^2+^ to the skeletal muscle mitochondria and allowed us to estimate the [Ca^2+^] reached inside their matrix even considering differences among fibre types. Research may now be focused on the mechanisms of muscle mitochondrial Ca^2+^ exit.

### 3.3 Store-operated Ca^2+^ entry in skeletal muscle

SOCE refers to a Ca^2+^ influx activated in response to the SR depletion and functions in most cells to refill these stores. Since skeletal muscle ECC is independent of extracellular Ca^2+^ (Section 2.2), the interest in SOCE was low for more than 15 years after its discovery. However, the demonstration of a Ca^2+^ influx in response to acute SR depletion (Kurebayashi and Ogawa, 2001) independent of any I_Ca_, opened a bulk of work addressing the question of the nature and physiological role of this Ca^2+^ influx in skeletal muscle. It was proposed that its function could be important in muscle growth, development and contractile function, as well as SR refill to delay fatigue (Stiber et al., 2008; Wei-LaPierre et al., 2013; Sztretye et al., 2017; Michelucci et al., 2018). However, a great depletion of SR may not be necessary to activate SOCE, since it is rapidly activated in response to a single AP (Koenig et al., 2018). Here we will present the main facts that conducted to these conclusions and a brief historical description of this mechanism, to understand its relevance in skeletal muscle.

#### 3.3.1 Basic concepts

SOCE was first described in non-muscle cells, where the depletion of intracellular Ca^2+^ stores in the continuous presence of inositol triphosphate induced the so called “capacitative Ca^2+^ entry” (Putney, 1986). That entry was a small rectifying highly Ca^2+^ selective current, not affected by Ca^2+^ channel blockers (Hoth and Penner, 1992) called Ca^2+^ Release Activated Ca^2+^ Current (I_CRAC_). Two research groups then identified in 2005 the Stromal Interacting Molecule (STIM) as a single-pass transmembrane EF-hand protein that acts as Ca^2+^ sensor in the endoplasmic reticulum lumen of many cells with a low affinity of ∼200–600 μM (Liou et al., 2005; Roos et al., 2005; Zhang et al., 2005; Stathopulos et al., 2006; Canato et al., 2010; Friedrich et al., 2010). In 2006 it was confirmed the interaction of STIM with a protein in the plasma membrane called Orai, which constitutes the transmembrane pore of the CRAC complex (Vig et al., 2006b, 2006a; Feske et al., 2006; Prakriya et al., 2006; Soboloff et al., 2006; Hou et al., 2018). STIM-Orai complexes constitute the Ca^2+^ entry units (CEU). In mammals, two STIM genes, STIM1 and STIM2, and three Orai genes, ORAI1, ORAI2 and ORAI3, have been identified (Zhang et al., 2005; Vig et al., 2006b; Feske et al., 2006). More details about SOCE in non–excitable cells are given elsewhere (Prakriya and Lewis, 2015; Putney, 2017).

#### 3.3.2 Is Store-operated Ca^2+^ entry relevant to skeletal muscle?

Skeletal muscle highly expresses STIM1 and Orai1 (Stiber et al., 2008; Vig et al., 2008). Both can interact with other channels such as TRPC1 and RyR1 and form complexes that act as the store operated channels complex (Stiber et al., 2008). Also, a longer spliced variant of STIM1, STIM1L, is highly expressed in skeletal muscle, where it colocalizes with Orai1 and binds to actin, forming permanent clusters (Darbellay et al., 2011). The presence of this molecular machinery explains the existence of functional SOCE in adult fibres from skeletal muscle, as first described in mouse EDL bundles after depletion of the SR by repetitive exposure to high K^+^ in the presence of SERCA inhibitors (Kurebayashi and Ogawa, 2001).

The presence of SOCE in skeletal muscle was also confirmed in myotubes (Pan et al., 2002; Shin et al., 2003; Cherednichenko et al., 2004; Lyfenko and Dirksen, 2008; Stiber et al., 2008), in mechanically skinned rat EDL and soleus fibres (Launikonis et al., 2003; Launikonis and Ríos, 2007; Cully et al., 2016) and in mouse FDB enzymatically dissociated fibres (González Narváez and Castillo, 2007; Bolaños et al., 2009).

The presence of a permanent, actin stabilized coupling of STIM1-Orai1 at the triad may be puzzling, since it may not be necessary for the activation of a classical SOCE in skeletal muscle. Instead, this organization may be relevant to explain the SOCE rapid activation and deactivation associated with every single AP, which has been more recently characterized (Launikonis and Ríos, 2007; Edwards et al., 2010; Koenig et al., 2018; Koenig et al., 2019). This molecular organization may sense RyR-associated SR depletion microdomains, even when the bulk of the SR is not depleted. Neither I_Ca_ blockers nor Ca^2+^ buffers affect that rapid Ca^2+^ influx (Koenig et al., 2018). This fast-activated SOCE was named as phasic SOCE (pSOCE) to distinguish it from the slower activated, chronic SOCE (cSOCE) (Koenig et al., 2018; Koenig et al., 2019).

Other authors proposed that a triad SOCE pool permits the pSOCE and a second pool at the level of the longitudinal SR activates the cSOCE (Darbellay et al., 2011; Michelucci et al., 2019). This pool would activate slower after acute SR depletion under SERCA blocking and it could be involved in the CEUs formation after strenuous exercise (Boncompagni et al., 2017; Michelucci et al., 2019). It is still possible that the Orai in the elongated TT or the largely ignored longitudinal tubules, interact with the actin associated STIM1L at the I bands, accounting for the longitudinal SR activating slower SOCE. Another, controversial possibility, for the fast activation is that Orai1 would be activated by a direct conformational coupling to RyR and not to STIM1 (Lyfenko and Dirksen, 2008; Dirksen, 2009a).

In this context, Reddish and coworkers (Reddish et al., 2021) were able to follow local RyR1 Ca^2+^ release events at level of the jSR in mice FDB fibres expressing the low affinity genetically encoded Ca^2+^ dyes G-CatchER+ and/or CatchER + -JP45. They found that the jSR local Ca^2+^ release at RyR1 microdomains was 2.1-fold greater than global SR release with much faster kinetics than the depletion in the bulk SR and that if sensed by STIM1, could quickly activate SOCE locally (Reddish et al., 2021).

The existence of small, fast, repetitive tubular Ca^2+^ transients associated to SOCE is now convincing. However, its relevance to skeletal muscle function is not clear yet. Based on experimental data previously published, the maximum capacity of this mechanism was recently estimated to be between 3 and 70 nM Ca^2+^ for fibres type I and IIB, respectively, during a single twitch (Rincón et al., 2021). Remembering that the peak sarcoplasmic Ca^2+^ ranges from 7 to 30 µM and that the free SR Ca^2+^ is over 1 mM (Section 3.1), it is difficult to assess what the function of this negligible amount of Ca^2+^ would be: it is neither relevant for refilling the SR nor for sustaining the sarcoplasmic [Ca^2+^]. One possible explanation is that some errors in the quantitation of the process have arisen because of underestimation of [Ca^2+^]. For instance, as discussed (Section 3.1), a peak [Ca^2+^] of 0.2–1 µM in mammalian fibres (Launikonis et al., 2009; Koenig et al., 2018) is untrue, and an inaccurate TT [Ca^2+^] calibration was acknowledged when we compare papers in which estimated values of 100 µM (Launikonis et al., 2009) were updated to be over 1 mM (Cully et al., 2016). Another option, if all mentioned SOCE estimates turn true (then, ruling out any role in SR refilling or sarcoplasmic [Ca^2+^] maintenance), and that the rate of exchange of Ca^2+^ with the tubules is low (Lamboley et al., 2021), is that SOCE in skeletal muscle may be, under certain conditions, a custodian of the fibre total amount of Ca^2+^. Although complete models considering the internal equilibrium of Ca^2+^ have already been presented (Section 3.1), models regarding its external equilibrium remain a pending task. Thus, more quantitative efforts should be done to complement structural studies to better understand the role of SOCE in skeletal muscle. Also, some observations should be reproduced in more physiological models, such as intact fibres, before stronger conclusions about the importance of SOCE in skeletal muscle can be drawn.

The apparent small capacity of this mechanism may explain why, in contrast to non-muscle cells, it has not been possible to electrophysiologically record I_crac_ in intact skeletal muscle fibres (Allard et al., 2006). The expected small size of the currents and the complex structure and electrical properties of the skeletal muscle fibres further complicate this approach.

Two conditions may highlight the importance of SOCE in intact fibres, the exercise, and the absence of CASQ. Mice subjected to treadmill exercise showed tubule remodeling which helped the SOCE machinery assemble following acute exercise and disassemble during recovery (Boncompagni et al., 2017; Michelucci et al., 2019). Preassembled CEUs are occasionally observed in non-exercised muscle, about 2/100 μm^2^ in EDL and FDB fibres, while in calsequestrin-null or knockout fibres (nCASQ1), increased to 40 and 17/100 μm^2^ respectively, probably to compensate the reduced store Ca^2+^ content due to the absence of CASQ. The associated increase in Ca^2+^ influx by SOCE observed in these fibres, seems to maintain contractile activation in response to repetitive high frequency stimulation and resistance to fatigue (Michelucci et al., 2020). Unfortunately, the Ca^2+^ signals in these works were not calibrated, avoiding estimating the quantitative importance of SOCE in those results. An increased SOCE during exercise, a condition expected to increase the exchange of Ca^2+^ with the exterior, is compatible with the hypothesis according to which SOCE may be a keeper of the total amount of Ca^2+^ inside the fibre. Also, since CASQ null fibres show a reduced amount of total Ca^2+^ content (Lamboley et al., 2021), an increased SOCE activation can be reexplained as trying to avoid a further reduction of the fibre’s Ca^2+^ content.

It has been remarked that there may not be enough space at the triad to accommodate STIM1-Orai1 aggregates together with DHPR, RyR1, plus junctophilin, triadin, calsequestrin, etc. However, the fast activation and deactivation previously shown is consistent with preformed complexes STIM1-Orai1 at the triads, close to the RyR1 release channel, and with limited mobility. Also, given the RyR distribution, at least 40% of the space is free of RyR in the jSR (see for instance (Block et al., 1988; Saito et al., 1988; Chen and Kudryashev, 2020)), which may be occupied by the STIM1-Orai1 clusters, i.e., the latter being surrounded by DHPR-RyR clusters as we propose in Figure 1. Our model shows that the movement restrictions imposed by DHPR-RyR to STIM-Orai explain why the CEU have to be preassembled, and fixed, in the jSR-TT membranes. Future super-resolution studies may shed some light on the actual distribution of all these proteins in the triadic space.

Proteins located at the triadic region are known regulators of SOCE. RyR1 and STIM1 colocalize and Ca^2+^ microdomains close to it regulate the activation of SOCE locally at jSR level (Stiber et al., 2008; Reddish et al., 2021). Removal of the cytoplasmic amino terminal region of the foot portion of the RyR1 abolishes SOCE (Sampieri et al., 2005). Under resting conditions, healthy muscle fibres show a low RyR1 leak but increased RyR1 leak augments the bidirectional Ca^2+^ exchange with the TT and mitochondrial metabolism to preserve normal contractile function (Lamboley et al., 2021). This is supported by the fact that SOCE-dependent Ca^2+^ influx is diminished or inhibited in myotubes lacking RyR1 or blocking the Ca^2+^ release with 100 µM Ry or azumolene (Pan et al., 2002; Zhao et al., 2006; Yarotskyy and Dirksen, 2012). However, in acute or partial SR-Ca^2+^ depleted intact dissociated FDB fibres, the pretreatment with 50 μM Ry, prior to SOCE activation, enhanced and maintained SOCE activated and only stopped by removing extracellular Ca^2+^ or applying SOCE blockers such as 2-APB (Bolaños et al., 2013). It is possible that this apparent contradiction early highlighted the existence of different roles of RyR1 in SOCE. Differential RyR conformational changes may confer it a function either as leak sensor or SR depletion sensor. These changes may be inhibited, potentiated or somehow modified by large concentrations of Ry, as shown in other contexts (Paolini et al., 2004). In any case, RyR1 participates Ca^2+^ influx by SOCE, an observation which deserves further study.

Given its role inside the SR, CASQ1 is a straightforward candidate to be a SOCE regulator. FDB CASQ1 knock down (CASQ1-null) skeletal muscle fibres diminish SR Ca^2+^ content, favoring the formation of STIM1 aggregates and their interaction with Orai1 that significantly enhanced SOCE (Zhao et al., 2010; Michelucci et al., 2020). On the other hand, the interaction of STIM1 with CASQ1 prevents its association to Orai1 thus limiting SOCE (Zhang et al., 2016). On the contrary, overexpression of the full length CASQ1 reduced SOCE in myotubes (Shin et al., 2003; Zhao et al., 2010), confirming CASQ as a direct modulator of SOCE.

Triadin and junctophilins could also participate in SOCE regulation, as their knockout or knockdown present alterations in the triad structure and an increase in the TT-SR distance, which could diminish STIM1-Orai1 interaction and SOCE (Hirata et al., 2006; Oddoux et al., 2009; Li et al., 2010; van Oort et al., 2011). Indeed, FDB fibres exposed to hypotonic solutions that could increase that distance show SOCE inhibition (Bolaños et al., 2013).

The TRPC channels subfamily consists of seven isoforms (TRPC1-7), which are expressed in skeletal muscle, though there is controversy about TRPC5-7 (Vandebrouck et al., 2002; Krüger et al., 2008; Zanou et al., 2010; Saüc and Frieden, 2017). TRPC1, TRPC3 and TRPC4 reside in the sarcolemma, where they have been reported to associate with RyR1, STIM1, STIM1L, or Orai1, forming ternary or heteromeric complexes (Vandebrouck et al., 2002; Liao et al., 2007; Ong et al., 2007; Liao et al., 2008; Ong et al., 2016; Antigny et al., 2017; Choi et al., 2020).

TRPC1 was the first and most consistently found to somehow participate in SOCE (Ong et al., 2007). In fact, other authors as well proposed that the influx of Ca^2+^ through Orai1 channels depends on the recruitment of TRPC1 into the plasma membrane where it is activated by STIM1 (Oláh et al., 2011; Ambudkar et al., 2017). In human skeletal myotubes, TRPC1, TRPC4 and STIM1L interact to sustain Ca^2+^ entry *via* SOCE that helps to maintain repetitive Ca^2+^ transients and differentiation (Antigny et al., 2017). Also, TRPC1 would be important as mediator of the RyR involvement in SOCE regulation (Sampieri et al., 2005). However, opposite evidence has also been provided, since TRPC1^−/−^ mice presented almost normal SOCE, but with a decreased resistance to fatigue and lower Ca^2+^ transients than TRPC1^+/+^ fibres (Zanou et al., 2010) and contradictory outcomes about the effect of the overexpression of TRPC1 on SOCE have been observed (Ong et al., 2007; Oláh et al., 2011). The colocalization of TRPC members to the SOCE machinery is acknowledged. However, a precise mechanism by which TRPC members regulate SOCE is still lacking, they may constitute a Ca^2+^ permeable pathway activated by STIM in parallel to Orai, may be regulators of Orai-mediated Ca^2+^ entry, or both.

In summary, the current renaissance of SOCE studies doubtlessly demonstrated its existence in skeletal muscle and its activation under several conditions. Preassembled complexes and rapid responses are in tune with the main muscle specialization, i.e., its very fast Ca^2+^ handling ability. As in other subfields of the ECC, such as the sarcoplasmic peak [Ca^2+^](Section 3.1) or the mitochondria (Section 3.2), putting reliable numbers to SOCE and SOCE-associated phenomena, would help to better understand its relevance *in situ*. Given its apparent low importance as keeper of the SR and sarcoplasmic [Ca^2+^], the possibility that it is a guardian of the total Ca^2+^ inside a muscle fibre arises. Also, clarification of the mechanism of regulation by neighbor proteins (e.g., RyR, TRPC) is pending. Making clearer the structural organization of the SOCE core and accessory machinery would benefit from super-resolution techniques.

### 3.4 Pharmacology of the excitation–contraction coupling

Table 2 presents details about the most important molecules that have allowed to study mechanisms relevant to the ECC. It highlights the fact that many of them came out to be less specific than initially thought, making some conclusions in several papers untrustworthy. The search for new, better (e.g., highly specific, less toxic) compounds with ECC applications is utterly encouraged.

**TABLE 2:** Pharmacology of the ECC in skeletal muscle.

Compound	Concentration	Mechanism of action	Comments	References
DHPR^a^, Ca_v_ 1.1 antagonists				
D-600	10–50 μM	Blocks I_CaL_ and ECC	Favors contractile inactivation. Use dependent. Reversible.	Caputo and Bolaños, (1987), Caputo and Bolaños, (1989), Carney-Anderson et al. (1997)
Nifedipine	0.01–200 μM	Blocks I_CaL_, allosteric inhibitor, reversible	Blocks charge movement and SR Ca^2+^ release. Membrane voltage dependent effect. Different effects on twitches and K^+^-contractures, concentration dependent. At >20 µM is less specific and blocks other voltage-gated channels such as K^+^ channels.	Rios and Brum, (1987), Dulhunty and Gage, (1988), Zhao et al. (2019)
Nitrendipine	0.1–1 μM	Less effect on I_CaL_. Reversible	Blocks K^+^-contractures, not twitches, releases Ca^2+^ from RyR.	Frank, (1987), Lüttgau et al. (1987)
Diltiazem	1–100 μM	Blocks I_CaL_, pore blocker, reversible. Also blocks SERCA	Potentiates the twitch, lowers the mechanical threshold potential, causes paralysis	Walsh et al. (1986), Walsh et al. (1988), Lüttgau et al. (1987), Zhao et al. (2019)
Verapamil	1–100 μM	Blocks I_CaL_, pore blocker, reversible	Blocks twitches, contractures, and AP	Walsh et al. (1986), Frank, (1987), Zhao et al. (2019)
Cd^2+^, Ni^3+^	0.2–2 mM	Block I_CaL_		Walsh et al. (1986), Lüttgau et al. (1987), Mould and Dulhunty, (1999)
DHPR, Ca_v_ 1.1 agonists				
Bay K 8644	1–10 μM	Enhances I_CaL_	Potentiates the twitch	Oz and Frank, (1994), Weigl et al. (2000)
	<20 μM (↑)	Increases (↑) or decreases (↓) twitch	The effect on the twitch depends on concentration and activation pattern. Increases the mean open time of the Ca^2+^ channel.	Dulhunty and Gage, (1988), Williams and Ward, (1991), Zhao et al. (2019)
	>50 μM (↓)			
RyR antagonists				
Ryanodine	<10 μM	Induces SR Ca^2+^ release and a channel subconductance state	Binds to the RyR with very high affinity. Its binding to the RyR is increased in presence of Ca^2+^ (µM) and ATP. Prolongs the relaxation phase of twitch.	Meissner, (1986), Imagawa et al. (1987), Lattanzio et al. (1987), Lai et al. (1988), Xu et al. (1998), Bolaños et al. (2013), des Georges et al. (2016)
	≥50 µM	Inhibits the SR Ca^2+^ release and the channel open probability	Completely blocks the channel	
Ruthenium Red	5–30 μM	Inhibits the SR Ca^2+^ release and the channel open probability	Potentiates the twitch, prolongs the AP, locks the channel in the closed state, inhibits the Ca^2+^ loading of SR vesicles, inhibits the binding of ryanodine to the RyR. Inhibits mitochondrial Ca^2+^ uptake (see below).	Fleischer et al. (1985), Imagawa et al. (1987), Lai et al. (1988), Delbono and Kotsias, (1989), Xu et al. (1998)
Dantrolene	10–50 μM	Inhibits the SR Ca^2+^ release and reduces the open probability of the channel, only in presence of cofactors	Requires Mg^2+^, ATP and probably calmodulin as cofactors to directly inhibit the RyR. More effective in presence of low Ca^2+^ (<1 µM). Reduces twitch tension itself, also increases I_Na_.	Ebashi, (1976), Moulds, (1977), Caputo, (1983), Caputo and Bolaños, (1987), Krause et al. (2004), Oo et al. (2015), Diszházi et al. (2019), Sarbjit-Singh et al. (2020)
Tetracaine	0.2–1 mM	Inhibits the SR Ca^2+^ release and the channel open probability	Abolishes Q^Ὑ^ component of charge movement, blocks Na^+^ channels	Caputo, (1983), Xu et al. (1993), Xu et al. (1998), Csernoch et al. (1999)
Procaine	3–10 mM	Reduces the SR Ca^2+^ release and the channel open probability	Does not shift sensitivity of the RyR to Ca^2+^. Reduces the AP, blocks Na^+^ channels. Reduces the contraction. pH dependent	Caputo, (1983), Xu et al. (1993), Ogawa et al. (1999)
RyR1 agonists				
Caffeine	μM to 10 mM	Increases the open probability of the channel and the SR Ca^2+^ release	Makes the Ca^2+^ release more sensitive to Ca^2+^ (μM). Lowers the mechanical threshold. Potentiates twitch. Downregulates murine skeletal muscle Na_v_1.4 function. Reversible. Its analog pentifylline is more potent.	Axelsson and Thesleff, (1958), Caputo, (1983), Xu et al. (1998), des Georges et al. (2016), Liu et al. (2021), Reggiani, (2021)
4-CmC	0.05–1 mM	Increases the open probability of the channel and the SR Ca^2+^ release	Potent and reversible	Herrmann-Frank et al. (1996), Westerblad et al. (1998), Bolaños et al. (2009)
4-CEP	20–500 μM	Increases the SR Ca^2+^ release	More potent than 4-CmC and Caffeine. Reversible	Westerblad et al. (1998)
Doxorubicin	1–100 μM	Increases the SR Ca^2+^ release	Used in skinned fibres.	Zorzato et al. (1985)
Imperatoxin A	10–50 nM	Opens the RyR in a long subconductance state	Increases the duration of sparks.	Tripathy et al. (1998), Shtifman et al. (2000), Dulhunty et al. (2004)
SERCA blockers				
Cyclopiazonic Acid	1–10 μM	Reversible SERCA blocker	Upregulates murine skeletal muscle Na_v_1.4 function	Seidler et al. (1989), Capote et al. (2005), Calderón et al. (2014a), Liu et al. (2021)
Thapsigargin	0.1–10 μM	Irreversible SERCA blocker	Potent SERCA inhibitor by favouring the E2 conformation, which reduces the affinity for Ca^2+^	Kijima et al. (1991), Wictome et al., 1992a, 1992b
BHQ, also known as TBQ	0.1–30 μM	Reversible SERCA blocker	SERCA inhibitor by favouring the E2 conformation, which reduces the affinity for Ca^2+^. Does not alter the Ca^2+^ sensitivity of the contractile apparatus. In heart, BHQ at >10 μM affects Ca^2+^ and K^+^ currents, but this has not been investigated in skeletal muscle.	Wictome et al. (1992b), Westerblad and Allen, (1994), Miller et al. (2015)
Mitochondria				
FCCP	0.2–2 μM	Proton ionophore which collapses the mitochondrial potential	Inhibits mitochondrial Ca^2+^ uptake. Induces concentration and time-dependent cell death.	Bolaños et al. (2008), Bolaños et al. (2009), Caputo and Bolaños, (2008), Zhou et al. (2010), Calderón et al. (2014a)
Ru360	200 nM-30 μM	Specific blocker of the MCU	Inhibitor of mitochondrial Ca^2+^ uptake	Emerson et al. (1993), Matlib et al. (1998), Zhou et al. (2010), Calderón et al. (2014a), Kirichok et al. (2004)
Ruthenium red	200 nM-50 μM	Inhibitor of mitochondrial Ca^2+^ uptake	Also, inhibitory effects on RyR1 and other cellular processes (see above).	Matlib et al. (1998), Kirichok et al. (2004)
NCX blockers				
KB-R7943	10–20 μM	Reverse NCX mode blocker. Reversible, non-specific.	Also inhibits SOCE, RyR and MCU. Reduces fibre excitability and reduces Ca^2+^ transients amplitude (see below).	Iwamoto et al., 1996, 2007, Iwamoto and Shigekawa, (1998), Arakawa et al. (2000), Niu et al. (2007), Santo-Domingo et al. (2007), Barrientos et al. (2009), Calderón et al. (2014a)
SN-6	2–10 μM	Reverse NCX mode blocker. Reversible	It seems not to alter fibre excitability. Reversible	Iwamoto et al. (2007), Niu et al. (2007), Barrientos et al. (2009), Calderón et al. (2014a)
DCB	10–30 μM	Forward NCX mode inhibitor. Reversible		Curtis, (1988), Calderón et al. (2014a)
Contraction uncouplers				
BDM	2–20 mM	Contraction uncoupler by affecting the force generating step in the crossbridge cycle.	May affect Ca^2+^ transients amplitude and reduce the Ca^2+^ sensitivity of the contractile apparatus	Fryer et al. (1988), Horiuti et al. (1988), Mckillop et al. (1994), Lyster and Stephenson, (1995), Capote et al. (2005), Iwamoto, (2018)
BTS	20–50 μM	Inhibits myosin ATPase activity and weakens actomyosin interaction, affecting the force generating step of the crossbridge cycle	Specific to the skeletal myosin heavy chain II. Eliminates movement artifacts in Ca^2+^ transients. Does not affect fluorescence transients amplitude	Cheung et al. (2002), Caputo and Bolaños, (2008), Calderón et al. (2009), Iwamoto, (2018)
Blebbistatin	0.5–5 μM	Inhibits myosin II ATPase by affecting the force generating step of the crossbridge cycle	Acts on cardiac, skeletal, and smooth muscle and non-muscle myosin II. Light sensitive and phototoxic	Limouze et al. (2004), Iwamoto, (2018), Roman et al. (2018)
Contractile potentiators Type A (Lower the contractile threshold)				
SCN^−^	20 mM	Potentiates twitch	Lowers the contractile threshold. Prolongs the AP	Hodgkin and Horowicz, (1960b), Mashima and Matsumura, (1962), Moulds, (1977), Caputo, (1983), Calderón et al. (2014b)
NO_3_^−^	Substitutes Cl^−^	Potentiates twitch	Prolongs the mechanically effective period. Reduces the contractile threshold.	Hodgkin and Horowicz, (1960b), Mashima and Matsumura, (1962), Caputo, (1983), Calderón et al. (2014b), Caputo et al. (2016)
ClO_4_^−^	10 mM	Potentiates twitch	Shifts the activation curve towards more negative potentials. Lowers the AP threshold.	Gomolla et al. (1983), González and Ríos, (1993), Calderón et al. (2014b), Caputo et al. (2016)
Contractile potentiators Type B (Prolong the action potential)				
Zn^2+^	0.05–1 mM	Potentiates twitch	Increases the AP duration	Isaacson and Sandow, (1963), Taylor et al. (1972), Caputo, (1983), Caputo et al. (2016)
Cd^2+^	1–1.5 mM	Potentiates twitch	Increases the AP duration and overshoot. Blocks I_CaL_	Mould and Dulhunty, (1999)
Mn^2+^	1 mM	Potentiates twitch	Increases AP threshold. Prolongs the AP. Alters mechanical threshold.	Chiarandini and Stefani, (1973), Ebashi, (1976), Caputo, (1983)
	≥10 mM	Decreases twitch and K^+^-contractures		
Contractile potentiators (Others)				
DES	5–10 μM	Potentiates twitch	Does not affect AP. Blocks SERCA. Slows rise and decay phase of twitch and tetanus	Khan, (1979), Caputo et al. (2016)
DAP	0.3–1 mM	Highly potentiates twitch	Blocks K^+^ channels. Slows the AP repolarization. Slows rise and decay phase of twitch and tetanus	van Lunteren et al. (2001); Ionno et al. (2008), Bolaños et al. unpublished results
Adrenaline, Terbutaline, Isoprenaline	0.1–30 μM	β-agonists, increase SR Ca^2+^ release	Positive inotropic and lusitropic effects	Cairns and Borrani, (2015)
SOCE blockers				
2-APB	≥30 μM	Blocks SOCE by inhibiting Orai1 and STIM-Orai interaction. Reversible, non-specific	Also inhibits IP_3_ receptor and other channels depending on concentration. Reduces Q^Ὑ^ component of charge movement. Inhibits I_CaL_. At <20 μM can enhance Orai3 function	Bolaños et al. (2009), Olivera and Pizarro, (2010), Putney, (2010), Wei et al. (2016)
DPB162-AE	40–200 nM	Blocks SOCE. Reversible	More specific 2-APB analog	Goto et al. (2010), Putney, (2010)
SKF-96365	100 μM	Non-specific SOCE inhibitor	Inhibits SR Ca^2+^ release, I_CaL_ and charge movement. Reversible	Jan et al. (1999), Olivera and Pizarro, (2010)
KB-R7943	10 μM	Non-specific SOCE inhibitor	Also inhibits NCX and affects other cellular processes (see above)	Arakawa et al. (2000)
BTP2	5–10 μM	Orai1 inhibitor	Indirectly affects electrically evoked SR Ca^2+^ release in skinned fibres, an effect not seen in intact FDB fibres exposed to 10 µM for up to 25 min, demonstrating a limited diffusion to the myoplasm.	Li et al. (2010), Meizoso-Huesca and Launikonis, (2021), Wei-LaPierre et al. (2022)
La^3+^	0.1–1 μM	Potent and relatively specific	Blocks also I_Ca_, and ECC	Bird et al. (2008), Putney, (2010)
Gd^3+^	≤5 μM	SOCE inhibitor	Specific at low concentration, if ≥ 100 μM blocks I_Ca_ and PMCA	Bird et al. (2008), Putney, (2010)
Others				
Tetrodotoxin	1–100 nM	Blocks Na^+^ channels and AP		Ebashi, (1976), Catterall, (1980)
Heparin	0.1–0.2 mg/ml	Potentiates twitch and tetanic tension	Prolongs the AP	Lamb et al. (1994), Martínez et al. (1996)
High K^+^	>50 mM	Depolarizes the sarcolemma	Activates the ECC	Hodgkin and Horowicz, (1959), Hodgkin and Horowicz, (1960a), Winegrad, (1970), Caputo and Bolaños, (1994)
Digoxin and Ouabain	0.05–1 μM and 0.1–1 μM	Na^+^/K^+^ ATPase blockers	Increase Ca^2+^ transients and tension. Potentiate SR Ca^2+^ release.	Sárközi et al. (1996)

a AP: action potential; 2-APB: 2-aminoethyldiphenyl borate; ATP: adenosine triphosphate; BDM: 2,3-butanedione 2-monoxime; BHQ: 2,5-di(tert-butyl)-1,4-benzohydroquinone; BTS: N-benzyl-ptoluene sulphonamide; BTP2: N-{4-[3,5-bis(Trifluoromethyl)-1H-pyrazol-1-yl]phenyl}-4-methyl-1,2,3-thiadiazole-5-carboxamide; 4-CEP: 4-chloro-3-ethylphenol; 4-CmC: 4-Chloro-m-Cresol; CICR: Ca^2+^-induced Ca^2+^ release; DAP: 3,4-diaminopyridine; DCB: 2´-4´ dichlorobenzamil hydrochloride; DES: diethylstilbestrol; DHPR: dihydropyridine receptors; DPB162-AE: diphenyl borate 162-AE; ECC: excitation-contraction coupling; FCCP: Carbonyl cyanide-p-trifluoromethoxyphenylhydrazone; I_CaL_: L-type Ca^2+^ current; KB-R7943: 2-[4-[(4-nitrophenyl)methoxy]phenyl]ethyl ester carbamimidothioic acid methanesulfonate; MCU: mitochondrial Ca^2+^ uniporter; NCX: Na^+^/Ca^2+^ exchanger; PMCA: plasma membrane Ca^2+^ adenosine triphosphatase; Ru360: Oxo-bridged dinuclear ruthenium amine complex; RyR: ryanodine receptor; SKF-9635: 1-[beta-[3-(4-methoxyphenyl)propoxy]-4-methoxyphenethyl]-1H-imidazole hydrochloride; SN-6: 2-[[4-[(4-Nitrophenyl)methoxy]phenyl]methyl]-4-thiazolidinecarboxylic acid ethyl ester benzyloxyphenyl; SOCE: store-operated Ca^2+^ entry; SR: sarcoplasmic reticulum: STIM-Orai: store operated machinery.

### 3.5 Emerging topics

#### 3.5.1 Super-resolution advances and excitation–contraction coupling pioneering studies

Including stimulated emission depletion (STED), structured illumination microscopy (SIM), photo-activated localization microscopy (PALM), stochastic optical reconstruction microscopy (STORM) and their modifications, super-resolution is a novel technique with enormous potential to study skeletal muscle and help solve some issues in ECC. Membrane rearrangements and clustering of proteins, under different experimental conditions, would suitably be studied with up to a 20 nm lateral resolution in fixed and living cells (Jayasinghe et al., 2015; Mishin and Lukyanov, 2019). However, it has been underexploited, probably because of the high costs and still low availability of super-resolution equipment in muscle laboratories.

Monitoring the morphology of the neuromuscular junction using SIM and STORM allowed to propose a new model in which the ACh receptor (AChR) is not located all over the postsynaptic membrane but restricted to the area surrounding the opening of junctional folds (York and Zheng, 2017). Also, STED images showed that width of crests and distance between them with AChR become altered in some neuromuscular diseases (Marinello et al., 2021).

The study of the subsarcolemmal tubular system with STORM revealed an enrichment in longitudinal tubules and branches in different directions (Jayasinghe et al., 2015), being the structural basis of the synchronization of membrane excitation with Ca^2+^ release from the SR with a safety factor, and probably supporting part of the remodeling potential of the tubular system involved in SOCE. Resolution and quality improvements for STORM images acquisition and ensemble allow to see the boundary membranes and the lumen of the TT (Sun et al., 2014), opening the door for future functional studies restricted to nanoregions.

The approach of pioneers using STORM to see the nanoscale organization of the RyR and CASQ triadic proteins (Jayasinghe et al., 2014), may be extended to simultaneously observe the DHPR-RyR and the STIM-Orai-TRPC clusters. Calculating distances and protein densities in nano- or micro-areas *in situ*, in different fibre types, would generate a more comprehensive picture of the triadic space. Also, increases in temporal resolution are expected to join the high spatial resolution of nanoscopy to see Ca^2+^ microdomains, useful for instance in SOCE and mitochondria studies.

#### 3.5.2 Excitation–contraction coupling in induced pluripotent stem cells-derived muscle cells

Notable work demonstrated that mature mammalian cells can be dedifferentiated to render induced pluripotent stem cells (iPSC), which in turn could be redifferentiated to almost any cell of the three germ layers (Takahashi and Yamanaka, 2006; Takahashi et al., 2007). As an example of the potential of iPSC to be differentiated to mesodermic cell types, the authors demonstrated for the first time the generation of iPSC-derived muscle tissue (Takahashi et al., 2007).

iPSC-derived skeletal myocytes obtained in 2D cultures have AP with pretty much the same kinetics as mature muscle fibres. However, although responsive to K^+^ depolarization in a way non-dependent on external Ca^2+^, their ECC is very immature, from structural and functional points of view (Skoglund et al., 2014; Lainé et al., 2018). Improved, 3D cultures of induced skeletal muscle bundles showed a greater degree of ECC differentiation, with sizable Ca^2+^ transients in response to electrical or ACh stimulation (Rao et al., 2018). Nevertheless, the fact that these signals are very slow suggests that the ECC machinery does not reach the maturity of adult muscle fibres in these preparations.

This knowledge, along with its associated technical developments, generates a model suitable for multiple applications in biomedical studies (Andrysiak et al., 2021). Envisioned applications include the understanding of physiological and pathophysiological events and search for new drugs tackling muscle diseases. Although great advances in the methodological protocols have generated quasi-mature muscle cells from iPSC, work is still needed to have fully differentiated fibres to exploit its potential for ECC studies. Also, efforts should be done to have absolute, rather than qualitative (presence or not) or relative (fold change), values of Ca^2+^ and ECC related variables in iPSC-derived muscle fibres.

## 4 Conclusion

We have presented the most complete picture of the ECC up to now. Seven decades of exciting research have identified a lot of proteins involved in ECC and a plenty of Ca^2+^ routes generated by those proteins. Assigning reliable numbers to some of those routes has also been successful. Nonetheless, as of today, two big issues await clarification: what is the exact mechanism of DHPR-RyR coupling and what are the details of the external equilibrium of Ca^2+^ during ECC. Quantitative approaches, emerging techniques such as super-resolution and iPSC, and finer pharmacology through more specific drugs, may help audacious researchers obtain the answers, hopefully sooner than later.
